# Insight into the Antibacterial Action of Iodinated Imine, an Analogue of Rafoxanide: a Comprehensive Study of Its Antistaphylococcal Activity

**DOI:** 10.1128/spectrum.03064-22

**Published:** 2023-04-26

**Authors:** Martin Krátký, Klára Konečná, Ondřej Janďourek, Adéla Diepoltová, Pavlína Vávrová, Barbora Voxová, Marcela Vejsová, Pavel Bárta, Szilvia Bősze

**Affiliations:** a Charles University, Faculty of Pharmacy in Hradec Králové, Department of Organic and Bioorganic Chemistry, Hradec Králové, Czech Republic; b Charles University, Faculty of Pharmacy in Hradec Králové, Department of Biological and Medical Sciences, Hradec Králové, Czech Republic; c Charles University, Faculty of Pharmacy in Hradec Králové, Department of Biophysics and Physical Chemistry, Hradec Králové, Czech Republic; d ELKH-ELTE Research Group of Peptide Chemistry, Budapest, Hungary; Keck School of Medicine of the University of Southern California

**Keywords:** antibiofilm activity, antistaphylococcal activity, *in vivo* toxicity evaluation, iodinated imine, methicillin- and vancomycin-resistant *Staphylococcus aureus*

## Abstract

In this study, we have focused on a multiparametric microbiological analysis of the antistaphylococcal action of the iodinated imine BH77, designed as an analogue of rafoxanide. Its antibacterial activity against five reference strains and eight clinical isolates of Gram-positive cocci of the genera Staphylococcus and *Enterococcus* was evaluated. The most clinically significant multidrug-resistant strains, such as methicillin-resistant Staphylococcus aureus (MRSA), vancomycin-resistant S. aureus (VRSA), and vancomycin-resistant Enterococcus faecium, were also included. The bactericidal and bacteriostatic actions, the dynamics leading to a loss of bacterial viability, antibiofilm activity, BH77 activity in combination with selected conventional antibiotics, the mechanism of action, *in vitro* cytotoxicity, and *in vivo* toxicity in an alternative animal model, Galleria mellonella, were analyzed. The antistaphylococcal activity (MIC) ranged from 15.625 to 62.5 μM, and the antienterococcal activity ranged from 62.5 to 125 μM. Its bactericidal action; promising antibiofilm activity; interference with nucleic acid, protein, and peptidoglycan synthesis pathways; and nontoxicity/low toxicity *in vitro* and *in vivo* in the Galleria mellonella model were found to be activity attributes of this newly synthesized compound. In conclusion, BH77 could be rightfully minimally considered at least as the structural pattern for future adjuvants for selected antibiotic drugs.

**IMPORTANCE** Antibiotic resistance is among the largest threats to global health, with a potentially serious socioeconomic impact. One of the strategies to deal with the predicted catastrophic future scenarios associated with the rapid emergence of resistant infectious agents lies in the discovery and research of new anti-infectives. In our study, we have introduced a rafoxanide analogue, a newly synthesized and described polyhalogenated 3,5-diiodosalicylaldehyde-based imine, that effectively acts against Gram-positive cocci of the genera Staphylococcus and *Enterococcus*. The inclusion of an extensive and comprehensive analysis for providing a detailed description of candidate compound-microbe interactions allows the valorization of the beneficial attributes linked to anti-infective action conclusively. In addition, this study can help with making rational decisions about the possible involvement of this molecule in advanced studies or may merit the support of studies focused on related or derived chemical structures to discover more effective new anti-infective drug candidates.

## INTRODUCTION

The loss of control over antibiotic-resistant bacteria has become a global issue due to severe and often untreatable bacterial infections. The consequences are reflected mainly as more complicated treatments, higher health care costs, and greater mortality (1, 2). Therefore, there is a crucial need for action to avert the threat of a developing global health care crisis. The discovery of new anti-infective compounds targeting especially resistant bacterial entities represents one of the options for combating this phenomenon.

Bacterial agents can use many protective mechanisms against the action of antimicrobial compounds. One of the protection strategies lies in biofilm formation (3, 4). Biofilms are complex microbial consortia adhering to inert or living surfaces, embedded in an extracellular matrix, which provides its cell participants a protective shield against various hostile environmental factors (sanitizers, antibiotics, UV damage, desiccation, and host immune defense, etc.) (5, 6). It is currently stated that approximately 80% of all bacterial infections are associated with biofilm formation in the host environment, and in general, these biofilm-associated infections are accompanied by a highly recalcitrant, recurring, chronic course of infectious disease (7–9).

Some of the most common pathogens mentioned in the context of antibiotic resistance are bacteria of the genus Staphylococcus. The most frequently mentioned pathogens are methicillin-resistant Staphylococcus aureus (MRSA) and vancomycin (VAN)-resistant S. aureus (VRSA) strains (10, 11). MRSA strains are also classified as multidrug-resistant strains, called “superbugs.” These strains have emerged and show resistance to conventional antibiotics from different groups, e.g., fluoroquinolones, aminoglycosides, and macrolides, etc. (12). The emphasis placed on the clinical significance of this pathogen is demonstrated by the declaration made by the World Health Organization identifying antibacterial research against MRSA as one of its priorities (13).

Staphylococci generally represent a challenging group of pathogens that are capable of causing both less severe and life-threatening infections. In addition, staphylococci are recognized as the most frequent causative agents of biofilm-associated infections such as endocarditis, osteomyelitis, and catheter-related bloodstream infections (14, 15).

Another group of bacteria often mentioned within the context of the resistance phenomenon is represented by members of the genus *Enterococcus*. These bacteria are recognized as opportunistic pathogens that can cause life-threatening infections (urinary tract infections, bloodstream infections, and endocarditis, etc.) and also demonstrate a significant potential for the rapid acquisition of multidrug resistance determinants (16). The repertoire of anti-infective drugs to which they may be resistant is extensive and includes antimicrobials of last resort (linezolid, daptomycin, and tigecycline, etc.), acting against glycopeptide- and multidrug-resistant bacterial strains as well (17).

Imines (Schiff bases) have been investigated largely as potential antimicrobial agents (18, 19). We have recently designed, synthesized, and evaluated active antibacterial and antifungal imines derived from sulfonamide drugs, 4-aminobenzoic acid, and its analogues (20–23). Among these compounds, 3,5-dihalogenosalicylaldehyde-based imines showed promising antibacterial activity against Gram-positive bacteria, including MRSA. The derivatization of the salicylaldehyde-based imine scaffold by at least one iodine atom seems to be responsible for creating this greater antibacterial effect. The importance of this element for the antimicrobial properties of the developmental agents has been repeatedly reported (24–26). This is why we prepared and investigated a new compound, BH77, (*E*)-2-{[(4-chlorobenzyl)imino]methyl}-4,6-diiodophenol, an analogue of rafoxanide, as an agent to combat resistant Gram-positive cocci.

## RESULTS

### Chemistry.

**FIG 1:**
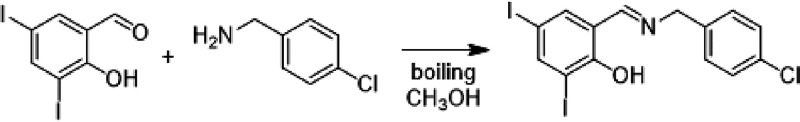
Synthesis and structure of BH77.

The novel title compound BH77, (*E*)-2-{[(4-chlorobenzyl)imino]methyl}-4,6-diiodophenol, was synthesized by a condensation reaction of 4-chlorobenzylamine with a mild excess of 3,5-diiodosalicylaldehyde in boiling methanol for 3 h, with an excellent yield of 95% (Fig. 1). This molecule was characterized by nuclear magnetic resonance (NMR) and infrared spectroscopy, and its purity was checked by melting point and elemental analysis measurements. *In silico* prediction of its physicochemical properties confirmed its favorable profile for drug-likeness (see Table S1 in the supplemental material).

### Evaluation of *in vitro* antibacterial efficacy against both reference strains and clinical isolates of the genera Staphylococcus and *Enterococcus*.

Based on preliminary studies, promising activity against some Gram-positive cocci was revealed. Therefore, to confirm these promising results, basic screening of antibacterial activity, including reference bacterial strains and conventional antibiotics such as ciprofloxacin (CIP), gentamicin (GEN), and vancomycin (VAN) (see Tables S2 and S3 in the supplemental material), was performed. In these assays, BH77 showed activity against bacteria of the genus Staphylococcus at concentrations ranging from 15.625 to 62.5 μM. Against another Gram-positive bacterium included in this screening, Enterococcus faecalis, activity corresponding to concentrations ranging from 62.5 to 125 μM was revealed. Within the range of the tested concentrations in the assays, no activity against Gram-negative bacteria was found.

Subsequently, an advanced, extended study of antibacterial efficacy, including medically relevant clinical bacterial strains with specified susceptibility profiles of the genus Staphylococcus and one strain of the genus *Enterococcus*, was performed (Table S4). To confirm the relevance of the results, MRSA, VRSA, and Staphylococcus epidermidis reference strains were included in the assays as well (Table 1).

**TABLE 1:** Antibacterial activity of BH77 against clinical isolates and reference strains of the genera Staphylococcus and *Enterococcus*^*a*^

Strain ID	Bacterial strain phenotype	MIC of BH77 (μM) determined by:		Biofilm production phenotype	Strain description
		Spectrophotometric detection (530 nm)	Visual detection		
131/16	MRSA	31.25	62.5	Moderate producer	Throat swab
136/16	MSSA	31.25	62.5	Moderate producer	Laryngeal swab
138/16	MRSA	31.25	62.5	Strong producer	Sputum
141/16	MSSA	31.25	31.25	Strong producer	Eye swab
ATCC 43300	MRSA	31.25	31.25	Weak producer	Reference laboratory strain ATCC 43300 (CCM 4750/NS)
153/16	MRSA	31.25	62.5	Moderate producer	Laryngeal swab
154/16	MSSA	31.25	62.5	Strong producer	Bronchoalveolar lavage fluid
198/16	VRE	31.25	31.25	ND	Catheter tube
ATCC 12228	SE	31.25	62.5	Strong producer	Reference laboratory strain ATCC 12228 (CCM 7844)
203/19 NIPH	VRSA	15.625	31.25	ND	NS, NIPH
CCM 1767	VRSA	15.625	15.625	ND	Reference laboratory strain CCM 7844

a The antibacterial action of compound BH77 was evaluated by the microdilution method according to EUCAST recommendations, with slight modifications. The MIC of BH77 was evaluated after 24 h by visual inspection and spectrophotometric measurement. For the categorization of biofilm production phenotypes, the crystal violet staining method was employed. Strain ID, internal laboratory identification number; ATCC, American Type Culture Collection; CCM, Czech Collection of Microorganisms, Czech Republic; MRSA, methicillin-resistant Staphylococcus aureus; MSSA, methicillin-sensitive Staphylococcus aureus; VRE, vancomycin-resistant Enterococcus faecium; VRSA, vancomycin-resistant Staphylococcus aureus; SE, Staphylococcus epidermidis; NS, not specified; ND, not determined; NIPH, National Institute of Public Health, Prague, Czech Republic.

BH77 showed antibacterial activity corresponding to an MIC of 31.25 μM (spectrophotometric detection) against clinical S. aureus and MRSA strains, which were recognized as moderate to strong biofilm producers in this study. The same activity was registered against the vancomycin-resistant Enterococcus faecium clinical isolate as well. For the other two included S. aureus strains that corresponded to the VRSA phenotype, more significant activity at an MIC of 15.625 μM was revealed (Table 1).

### Recognition of the bactericidal action, evaluation of the dynamics leading to the suppression of bacterial viability *in vitro*, and insight into target structures of bacterial cells affected by the action of BH77.

A microdilution method was employed to allow the recognition of the bactericidal or bacteriostatic action of BH77. MRSA ATCC 43300 was exposed to BH77 at various concentrations for 24 h. According to CFU calculations, the concentration of BH77 corresponding to the MIC led to a 99.89% reduction in bacterial viability compared to the initial inoculum. As presented in Table S5 in the supplemental material, a reduction of ≥99.9% after exposure to BH77 at concentrations corresponding to 2× to 8× MIC was registered. According to the above-mentioned criterion (Text S1.3), it can be stated that BH77 is a compound that displays bactericidal action. This result fully corresponds to the preliminary estimate of its antibacterial activity (Table S2), where no increase in the MIC after 48 h of incubation compared to the MIC corresponding to 24 h of incubation was registered.

**FIG 2:**
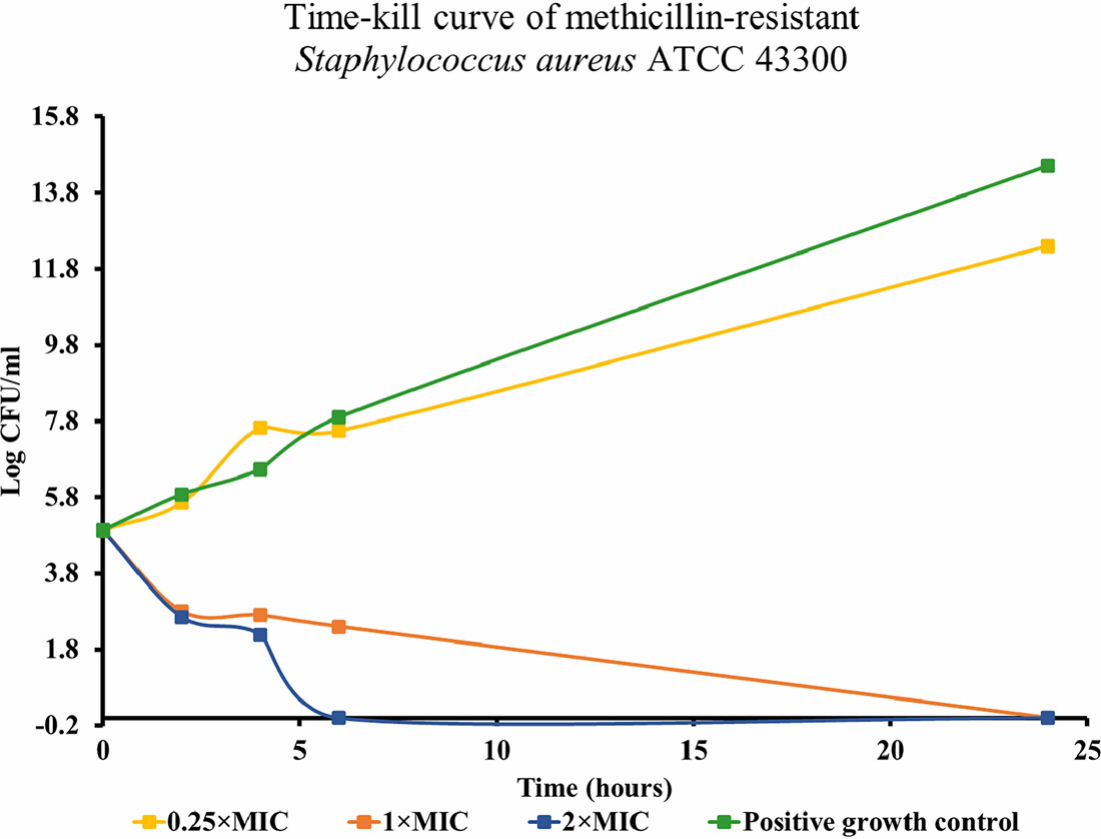
Time-kill curves of methicillin-resistant Staphylococcus aureus ATCC 43300 exposed to BH77 at different concentrations in relation to the respective multiples of the MIC over a period of 24 h. A positive control (bacterial suspension not affected by BH77) was included in the assays. Mean values of triplicate measurements of the CFU per milliliter and incubation times are plotted.

The bactericidal mode of action was also documented in time-kill studies. As part of the time-kill assays, both the viability and metabolic activity of the MRSA ATCC 43300 strain in response to different BH77 concentrations were mapped. The time-kill curve presented in Fig. 2 shows that already after 2 h, the number of viable bacteria (reflected in the total number of CFU) decreased due to the action of BH77 at 1× MIC and 2× MIC. At time intervals of 4 and 6 h, a relative stagnation in the number of CFU was registered after the exposure of bacteria to 1× MIC. After the exposure of bacteria to 2× MIC of BH77 for 6 h, viability was lost entirely. A 0.25× sub-MIC led to a slight increase in the CFU after a 4-h exposure. However, after 24 h, the number of CFU was partially reduced compared to the positive control.

**FIG 3:**
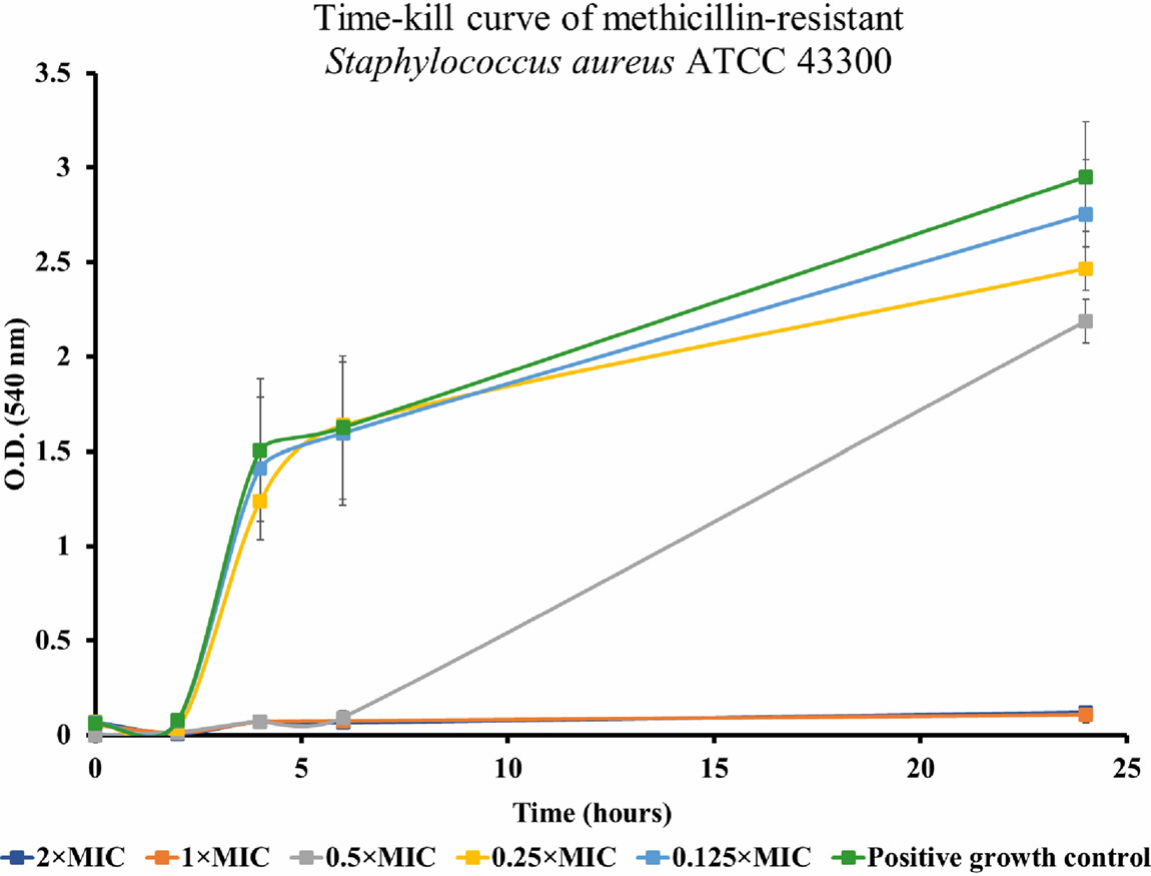
Time-kill curves of methicillin-resistant Staphylococcus aureus ATCC 43300 exposed to BH77 at different concentrations in relation to the metabolic activity detected by an MTT assay over a period of 24 h. A positive control (bacterial suspension not affected by BH77) was included in the assay. Mean values ± standard deviations (SD) of triplicate metabolic activity measurements and incubation times are plotted.

After the exposure of the bacterial strain to 1× MIC and 2× MIC of BH77, no metabolic activity was registered. After the exposure of bacteria to 0.5× sub-MIC, a suppression of metabolic activity within the first 6 h of interaction was registered. The metabolic activity of bacteria after interactions with 0.25× and 0.125× sub-MIC of BH77 for 6 h of incubation was not dramatically affected, and after 24 h, only a partial decrease in metabolic activity compared to the positive control was registered (Fig. 3).

### Profiling the *in vitro* interaction of BH77 with methicillin- and vancomycin-resistant Staphylococcus aureus strains and assessing the impact of selected conventional anti-infective drugs as potential partner compounds for BH77 in combination therapy.

A pharmacodynamic study was employed to perform an *in vitro* evaluation of the interaction of BH77 with three selected staphylococcal strains of the greatest clinical significance (MRSA and VRSA).

This study, based on the mapping of bacterial growth affected by the action of BH77, revealed that for all included strains, concentrations of BH77 corresponding to 31.25 and 15.625 μM led to the complete inhibition of the growth within a 20-h incubation interval (see Fig. S1 in the supplemental material). A concentration of 7.81 μM was not sufficient to suppress the growth of all bacterial strains. The growth of the MRSA ATCC 43300 strain affected by this subinhibitory concentration was reflected by the transition from lag to exponential phase after approximately 2.5 h of incubation, similar to the positive control (without any exposure to BH77). The whole growth profile correlated with the growth profile of the positive control, with the exception of a difference in the formation of a decreased bacterial biomass (Fig. S1A).

In both VRSA strains, a significant limitation of bacterial growth affected by a sub-MIC of 7.81 μM compared to the positive control was registered. Evidently, the activity of BH77 led to a growth delay reflected by a time shift (6.5 to 7 h) in the transition from lag to exponential phase in both strains (Fig. S1B and C). After a 20-h cultivation period, the growth of both strains was inhibited by more than 70% compared to the positive control. At a concentration of 3.906 μM, an analogous difference was recorded.

**FIG 4:**
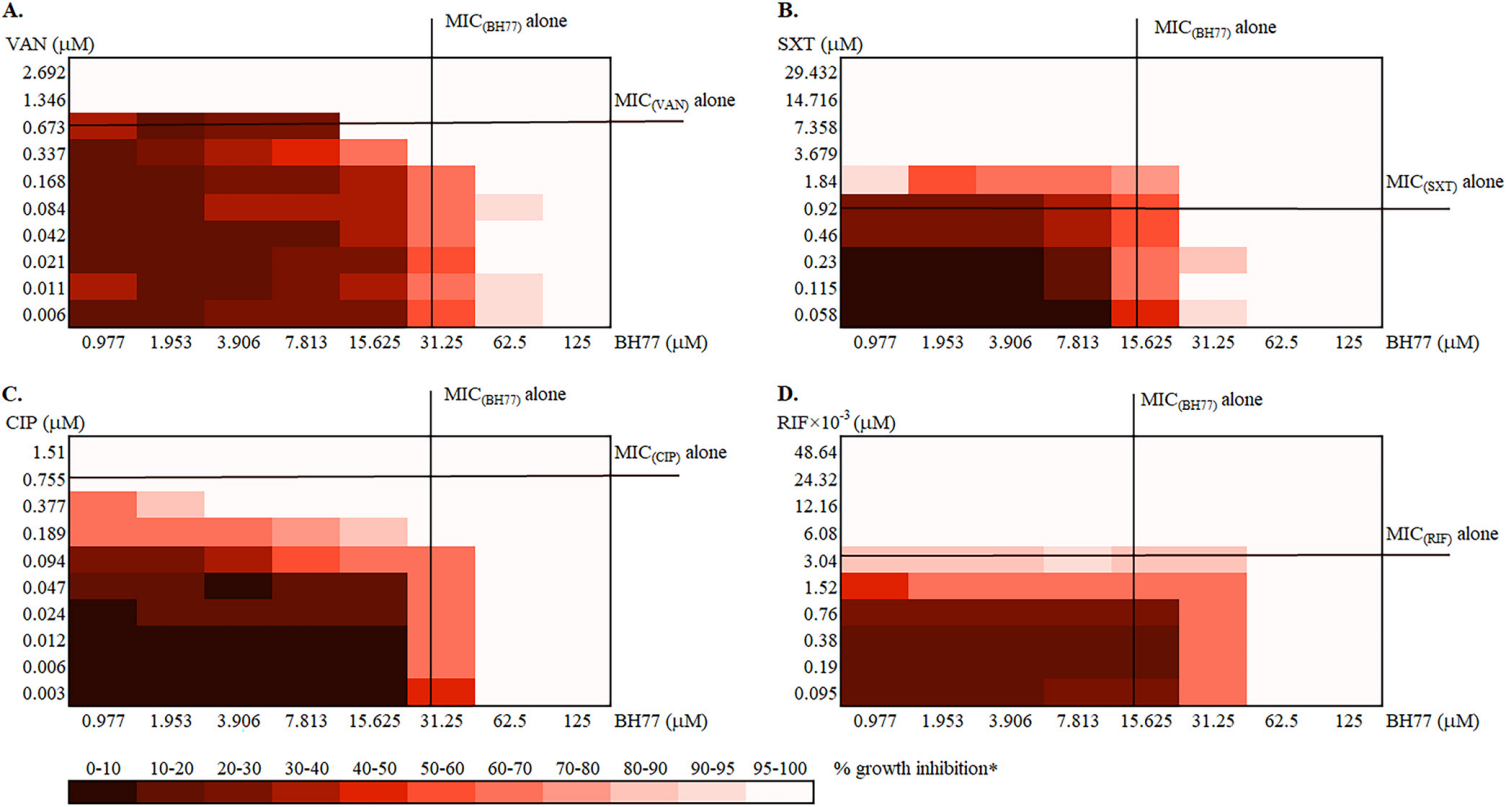
Two-color heat maps of checkerboard MIC tests. Heat plots of two-compound interactions of BH77 and vancomycin (VAN) (A), BH77 and trimethoprim-sulfamethoxazole (SXT) (B), BH77 and ciprofloxacin (CIP), and BH77 and rifampicin (RIF) (B), with methicillin-resistant Staphylococcus aureus strain ATCC 43300 show the percent inhibition of growth in comparison to a positive control (growth of bacteria not limited by the antibacterial action of the tested compounds) evaluated after 20 h of incubation. The MICs of the compounds alone correspond to an MIC_(VAN)_ of 0.673 μM and an MIC_(BH77)_ of 31.25 μM (A), an MIC_(SXT)_ of 0.92 μM and an MIC_(BH77)_ of 15.625 μM (B), an MIC_(CIP)_ of 0.775 μM and an MIC_(BH77)_ of 31.25 μM (C), and an MIC_(RIF)_ of 3.04 × 10^−3 ^μM and an MIC_(BH77)_ of 15.625 μM (D).

A checkerboard assay was used to test the activities of selected antistaphylococcal drugs with different mechanisms of action, namely, VAN, rifampicin (RIF), CIP, and trimethoprim-sulfamethoxazole (SXT), in combination with BH77. According to the total fractional inhibitory concentration (FIC) index (FICI) calculation, it was revealed that none of the above-mentioned compounds showed any synergic interaction in combination with BH77 (Fig. 4). A nonantagonistic effect was revealed with the combinations of VAN and BH77 (VAN+BH77) and CIP+BH77 at all concentration ratios (Fig. 4A and C and Table S6). An antagonistic effect was revealed for combinations of SXT+BH77 and RIF+BH77 at some concentration ratios (Fig. 4B and D and Table S6). As shown in Fig. 4B, BH77 appears to reduce the activity of SXT in an SXT+BH77 combination, and RIF reduces the activity of BH77 in a RIF+BH77 combination (Fig. 4D). In summary, from the selected antimicrobial drugs, CIP and VAN seem to be potential partner compounds for BH77 for combination (adjuvant) therapy targeting multiple vital bacterial processes (peptidoglycan, DNA, and protein synthesis).

### Insight into the mechanism of the action of BH77 reveals more potential cellular targets within bacterial cells.

This study employed a macromolecular synthesis assay and a membrane depolarization assay to investigate the potential bacterial cellular targets of the action of BH77. In the first above-mentioned assay, upon exposure to the tested compound, the percentage of radiolabeled precursors incorporated into biomacromolecules was measured. The results were compared with data obtained after treatment with known antibacterials (inhibitors of DNA, RNA, peptidoglycan, and protein synthesis). Basically, antimicrobial drugs can target one or more bacterial macromolecular synthesis processes (27). When evaluating BH77 (exposure of bacteria to 4× MIC of BH77), a significant cessation of [^3^H]leucine, [^3^H]thymidine, and [^3^H]*N*-acetylglucosamine incorporation was registered (Fig. S2). The greatest, statistically significant impact was registered for the protein synthesis pathway. A weaker but still significant effect was observed on the other two above-mentioned pathways, the peptidoglycan and DNA synthesis pathways. The inhibition of more macromolecular biosynthetic pathways can be observed with some antibacterial drugs (e.g., daptomycin) that bind to the bacterial membrane and lead to a loss of membrane potential (28). Based on this fact, a membrane depolarization assay was included in this study. In this assay, fluorometric measurement of membrane potential using a voltage-sensitive dye, 3,3′-dipropylthiadicarbocyanine iodide (DiSC_3_(5)), and chlorhexidine (CHX), as the positive control, was employed. As shown in Fig. S3 in the supplemental materials, compared to the negative control, only a slight increase in membrane depolarization was registered after the exposure of the bacteria to BH77 at a concentration corresponding to the minimum bactericidal concentration (MBC) (62.5 mM). The increase in membrane depolarization corresponded to only 14.31% compared to the CHX positive control (data normalized to the negative control).

### BH77 showed promising activity against biofilms produced *in vitro* by two clinically relevant staphylococcal strains.

Based on the revealed antibacterial activity of BH77 against reference strains together with biofilm-producing clinical isolates of the genus Staphylococcus, we also decided to evaluate the *in vitro* antistaphylococcal biofilm activity. Two biofilm-producing strains, methicillin-resistant Staphylococcus aureus ATCC 43300 and Staphylococcus epidermidis ATCC 1228, were used for MBIC (minimum biofilm inhibition concentration) and MBEC (minimum biofilm eradication concentration) determinations, which were evaluated using a microtiter plate biofilm assay. The conventional fluoroquinolone antibiotic CIP was included as a reference drug. As shown in Table 2, BH77 exhibited MBICs ranging from 125.0 to 250.0 μM (4 to 8 times higher than the MIC) for the S. aureus strain and 62.5 to 125 μM (2× to 4× MIC) for the S. epidermidis strain. The MBEC value, representing the concentration capable of eliminating or greatly reducing the number of living cells in preformed biofilms, corresponded to a 2-fold value of the MBIC for both strains (values 8 to 16 times higher than the MIC for the S. aureus strain and 4 to 8 times higher than the MIC for the S. epidermidis strain). In comparison, a conventional antistaphylococcal drug, CIP, exhibits MBIC activity corresponding to values 3 times higher than the MIC for the S. aureus strain and 1.5 to 3.0 times higher than the MIC for the S. epidermidis strain. However, noticeable increases in the MBECs, corresponding to a 127-fold increase in the MBIC for the S. aureus strain and a 254-fold increase in the MBIC for the S. epidermidis strain, were registered. As mentioned above, BH77 showed bactericidal activity. Therefore, it can be deduced that the lack of metabolic activity determined by evaluating the MBIC in bacterial cells released from preformed biofilms is coupled with a loss of bacterial viability linked to a decreased potential to limit the spread of infectious agents from infectious deposits known as biofilms. It is known that the resistance of bacteria in these consortia to antibacterial drugs can be increased up to 1,000 times compared to a planktonic form of growth (5, 29). Based on these findings and our results regarding the evaluated MBECs *in vitro*, it can be concluded that BH77 seems to be a promising antibiofilm compound.

**TABLE 2:** Evaluation of the antibiofilm activity of BH77 against two biofilm-producing staphylococcal strains, methicillin-resistant Staphylococcus aureus ATCC 43300 and Staphylococcus epidermidis ATCC 12228^*a*^

Type of activity	Value for bacterial strain	
	MRSA	SE
BH77		
MIC (μM)	31.25	62.5
MBIC (mg/L)	62.216–124.432	31.108–62.216
MBIC (μM)	125.0–250.0	62.5–125
Multiple of the MIC value	4–8×	2–4×
MBEC (mg/L)	124.432–248.863	124.432–248.863
MBEC (μM)	250–500	250–500
Multiple of the MIC value	8–16×	4–8×
CIP		
MIC (mg/L)	0.128	0.256
MBIC (mg/L)	0.381	0.381–0.763
Multiple of the MIC value	3×	1.5–3×
MBEC (mg/L)	48.8	97.6–195.3
Multiple of the MIC value	381.3×	381.3–762.9×

a The biofilm microtiter plate method was employed for minimum biofilm inhibition concentration (MBIC) and minimum biofilm eradication concentration (MBEC) evaluations.

### Assessment of BH77’s *in vitro* cytotoxicity and *in vivo* toxicity in an alternative animal model, Galleria mellonella.

To determine cytotoxicity *in vitro*, two human cell lines, namely, HepG2 and MonoMac6, were included in this study. The 50% inhibitory concentration (IC_50_) was used as an indicator of cytotoxicity. As shown in Fig. S4 in the supplemental material, the IC_50_ value determined for the HepG2 cell line was >100 μM, while that for the MonoMac6 cell line was >50 μM. Due to the limited solubility of BH77 in the appropriate cell line cultivation media, the IC_50_ cutoff values could not be determined. Nevertheless, these recognized *in vitro* cytotoxicity results did not rightfully eliminate the employment of the alternative animal model Galleria mellonella for the screening of *in vivo* toxicity. G. mellonella has been enjoying increasing popularity and is more often being used as a replacement for mice and rats in toxicity studies (30). Generally, the use of this model for toxicity testing has brought several benefits and seems to be fully appropriate for the discrimination of toxic and nontoxic chemicals (31).

**FIG 5:**
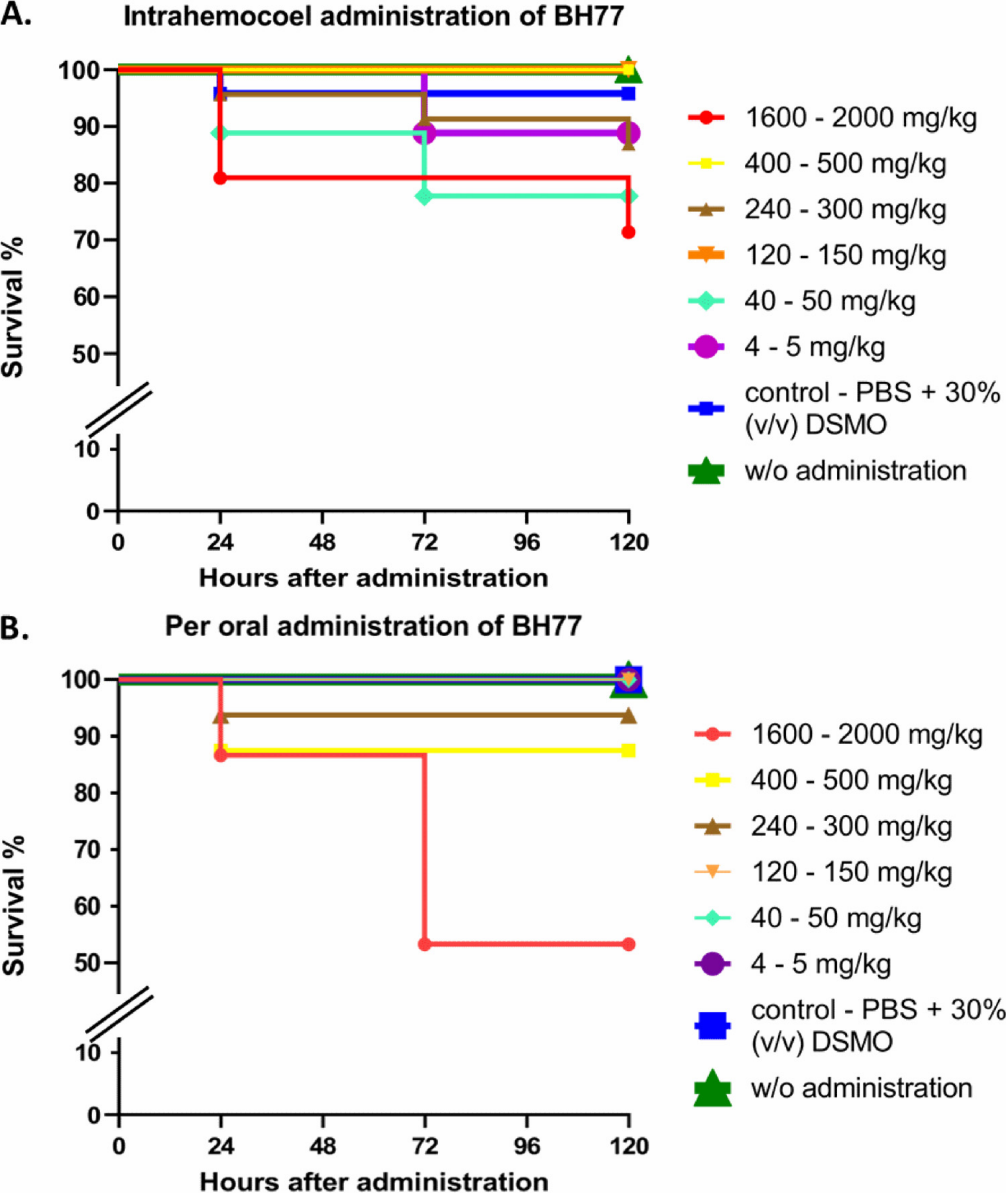
Survival curves of the Galleria mellonella animal model after intrahemocoel (A) and peroral (B) administration of BH77. After administration, larvae were incubated at 37°C for 5 days and inspected after 1, 24, 72, and 120 h of incubation.

Two ways of administering the tested compound into the animals were employed: injection into the hemocoel through the last left proleg and the peroral route of administration. The larvae were divided into groups according to the final concentration of the administered compound per kilogram of body weight of the animal. The doses corresponded approximately to the recommendations made in the Organisation for Economic Co-operation and Development test guidelines for chemicals (32). As shown in Fig. 5A and Table S7A in the supplemental material, in all groups after intrahemocoel administration, the LD_50_ (lethal dose leading to the death of 50% of the animals) was not reached. Therefore, it can be concluded that the LD_50_ of BH77 corresponds to a dose of >1,600 mg/kg of body weight of the larva. After peroral administration, in group 1 (with the highest administered dose, 1,600 to 2,000 mg/kg of body weight), a mortality rate near the LD_50_, precisely 46.67%, was observed (Fig. 5B and Table S7B). However, it is necessary to realize that for BH77, antibacterial activity toward the group of selected bacterial agents from the portfolio of medically significant human pathogens (Gram-positive cocci) was recognized, and an impact on the unique intestinal microbiome of this animal cannot be ruled out. Finally, all of these facts were reflected in the higher mortality rate after the peroral administration of BH77. Based on these findings, BH77 could be categorized into Globally Harmonized System class 4, which represents nontoxic or very-low-toxicity compounds (30).

Data from extended survival studies (groups with higher administered doses and control groups) were subjected to statistical analysis via pairwise comparisons using the log rank Mantel-Cox curve comparison test and the Mantel-Haenszel pairwise comparison test. After intrahemocoel administration, a significant effect of BH77 on the survival of larvae was registered after the data were analyzed via the pairwise log rank Mantel-Cox curve comparison test between the control group (30% [vol/vol] dimethyl sulfoxide [DMSO] in phosphate-buffered saline [PBS]) and group 1 (1,600 to 2,000 mg/kg of body weight) (χ^2^ = 4.935; *P* = 0.0263) and between group 1 (1,600 to 2,000 mg/kg of body weight) and group 2 (400 to 500 mg/kg of body weight) (χ^2^ = 7.478; *P* = 0.0062). Hazard ratios (Mantel-Haenszel pairwise comparison test) were calculated from increases in the BH77 concentration from 240 to 300 mg/kg of body weight to 400 to 500 and 16,00 to 2,000 mg/kg of body weight, corresponding to values of 0.1291 (95% confidence interval [CI], 0.01342 to 1.243) and 0.4135 (95% CI, 0.1062 to 1.610). After peroral administration, the data analyzed via the pairwise log rank Mantel-Cox curve comparison test revealed a significant effect between the control group (30% [vol/vol] DMSO in PBS) and group 1 (1,600 to 2,000 mg/kg of body weight) (χ^2^ = 9.315; *P* = 0.0023) and between group 1 (1,600 to 2,000 mg/kg of body weight) and group 3 (240 to 300 mg/kg of body weight) (χ^2^ = 5.921; *P* = 0.015). Hazard ratios (Mantel-Haenszel pairwise comparison test) were calculated from increases in the BH77 concentration from 240 to 300 mg/kg of body weight to 400 to 500 and 1,600 to 2,000 mg/kg of body weight, corresponding to values of 0.4903 (95% CI, 0.04724 to 5.090) and 0.1596 (95%CI, 0.03641 to 0.6998) (see Tables S8 and S9 in the supplemental material for all pairwise comparisons).

## DISCUSSION

Antibiotic resistance is one of the greatest threats to global health, with a potentially serious socioeconomic impact (33). Some public health organizations have described the rapid emergence of resistant infectious agents as a “crisis” that may have catastrophic consequences in the future (34). One of the strategies that might alleviate this serious prognosis lies in the support and encouragement of the discovery and research of new anti-infectives as well as the search for new or alternative approaches for the treatment of infections caused especially by multiresistant agents.

From the group of Gram-positive bacteria, a global pandemic of resistant S. aureus and *Enterococcus* species poses the greatest threat (35). For illustration, it was reported previously that superbugs of MRSA strains could be responsible for a higher mortality rate than, for example, HIV/AIDS or tuberculosis (34, 36).

In this comprehensive study, we have principally introduced the antistaphylococcal action of a newly synthesized compound designated BH77. This 3,5-diiodosalicylaldehyde-based imine can be considered an analogue of rafoxanide, a 3,5-diiodosalicylanilide anthelmintic that has recently been proposed for repurposing as an antibacterial agent (37).

To the best of our knowledge, no comprehensive study focused on the antistaphylococcal potential of newly synthesized iodinated imines has previously been published. To evaluate antibacterial activity, clinical strains were also employed. The introduction of these strains into *in vitro* anti-infective activity studies allowed us to confirm the previously evaluated promising activity and could also reflect the greater clinical relevance, mainly with regard to limited manipulation consisting only of handling with stock subcultures to avoid bacterial genetic and epigenetic changes.

In view of the fact that staphylococci are among the most medically significant pathogens causing infections associated with biofilm formation, we also focused our attention on screening the antibiofilm activity of BH77. Bacterial life in a biofilm community has a dynamic nature, and five stages of biofilm formation are recognized. The final step of the biofilm life cycle lies in biofilm dispersion, the detachment of cells and their reversion to a planktonic form. These liberated cells are then capable of disseminating in the host environment and establishing new biofilm communities as new reservoirs of infections (29). Therefore, it is also desirable to look closely at criteria recognizing the potential to limit the spread of infectious agents from infectious deposits known as biofilms. In our study, two parameters of antibiofilm action, the MBIC and MBEC, were evaluated. The first parameter reflects the inhibition of the metabolic activity of planktonic cells released from the complex bacterial biofilm consortium, and the second above-mentioned parameter is related to the loss of viability of biofilm-forming cells. According to the results obtained from the BH77 antibiofilm activity screening assay, it appeared that BH77 could be recognized as a promising antibiofilm compound. In addition, with regard to the revealed bactericidal action of BH77, it can be assumed that the loss of metabolic activity in released planktonic cells in the MBIC evaluation also reflects the loss of their viability, limiting their potential for the additional dissemination of infectious deposits.

One option to prevent the emergence and spread of resistance that might potentiate anti-infective attack lies in antimicrobial drug combination therapy (38). With this therapy, antimicrobial agents directed against multiple molecular targets or against multisubunit macromolecular machineries or structures are preferably selected. A combination of two or more drugs that exhibit cumulative efficacy, synergic effects, is strongly preferred. The synergic effect may additionally indicate reduced toxicity and adverse effects due to the lower therapeutic dose administered (39). From the portfolio of selected antibiotic drugs, CIP and VAN have been recognized at some concentration ratios with BH77 as suitable partner drugs for combination therapy. In fact, these two drugs hit the same bacterial targets, namely, DNA replication (CIP) and peptidoglycan synthesis (VAN), which were recognized by a macromolecular synthesis assay with BH77. This finding in no way reduces the importance at all. Although the main strategy of combination therapy is to inhibit targets in different biosynthetic pathways, combinations leading to the inhibition of different nodes in the same pathways or those that inhibit the very same target in a different way can be equally beneficial.

Finally, it should be emphasized that BH77 did not show any significant *in vitro* cytotoxicity or *in vivo* toxicity. For the purpose of assessing *in vivo* toxicity, the invertebrate model of Galleria mellonella larvae has been included. Galleria mellonella represents an alternative animal model that is increasingly being used as a replacement for mice and rats in infection and toxicity screening studies (30, 40). The use of this model for toxicity testing brings a number of benefits and seems to be fully appropriate for the discrimination of toxic and nontoxic chemicals (31). In conclusion, greater reliability of the toxicity of chemical compounds in mammals could be made by considering their toxicities in both cell cultures and G. mellonella larvae.

## MATERIALS AND METHODS

### Chemistry.

All of the reagents and solvents were purchased from Sigma-Aldrich (Darmstadt, Germany) and Penta Chemicals (Prague, Czech Republic), and they were used as received. The progress of the reaction and the purity of the product were monitored by thin-layer chromatography using a mixture of toluene and ethyl acetate (4:1, vol/vol) as an eluent. Plates were coated with 0.2-mm Merck 60 F254 silica gel (Merck Millipore, Germany) and visualized by UV irradiation (254 nm). The melting point (mp) was determined on a B-540 melting point apparatus (Büchi, Switzerland) using open capillaries. The reported values are uncorrected. Elemental analysis (C, H, and N) was performed on a Vario Micro Cube element analyzer (Elementar Analysensysteme, Germany). Both calculated and found values are given as percentages. The infrared spectrum (IR) was recorded on a Nicolet 6700 FT-IR spectrometer (Thermo Fisher Scientific, MA, USA) in the range of 600 to 4,000 cm^−1^ using an attenuated total reflectance technique. The nuclear magnetic resonance (NMR) spectra were measured in deuterated dimethyl sulfoxide (DMSO-*d*_6_) at ambient temperature on a Varian V NMR S500 instrument (500 MHz for ^1^H and 125 MHz for ^13^C; Varian Inc., Palo Alto, CA, USA). The chemical shifts, δ, are given in parts per million with respect to tetramethylsilane via signals of DMSO-*d*_6_ (2.49 for ^1^H and 39.7 for ^13^C spectra). The coupling constants (*J*) are reported in hertz.

The title compound BH77 was prepared as follows. 4-Chlorobenzylamine (2 mmol; 243.3 μL) was dissolved in 10 mL of methanol, and 3,5-diiodosalicylaldehyde (2.2 mmol; 822.6 mg) was then added gradually over 10 min with vigorous stirring. The reaction mixture was stirred at room temperature for 5 min and then refluxed for 3 h. The resulting suspension was stirred overnight at room temperature. After a 2-h storage at 4°C, the suspension was filtered off. The resulting crystals were washed successively with a small volume of a solution of cold methanol and diethyl ether and then dried to obtain pure BH77.

(*E*)-2-{[(4-Chlorobenzyl)imino]methyl}-4,6-diiodophenol (BH77): yellow solid; yield, 95%; mp, 174.5°C to 176°C. IR, 3,056, 1,624, 1,587, 1,492, 1,438, 1,417, 1,374, 1,283, 1,270, 1,158, 1,093, 1,039, 1,013, 868, 842, 816, 805, 737, 686, 674, 666, 654 cm^−1^. ^1^H NMR (500 MHz, DMSO-*d*_6_) δ 8.59 (1H, s, CH=N), 8.04 (1H, d, *J *= 2.2 Hz, H4), 7.76 (1H, d, *J *= 2.2 Hz, H6), 7.47 to 7.44 (2H, m, H3′, H5′), 7.41 to 7.38 (2H, m, H2′, H6′), 4.83 (2H, s, CH_2_). ^13^C NMR (126 MHz, DMSO) δ 165.82, 164.44, 148.67, 140.94, 136.46, 132.47, 130.03, 128.86, 118.54, 92.15, 77.43, 57.77. Analytically calculated for C_14_H_10_ClI_2_NO (497.50): C, 33.80; H, 2.03; N, 2.82. Found: C, 33.82; H, 2.05; N, 2.79.

### Advanced screening of antistaphylococcal and antienterococcal activity.

Clinical isolates of the genus Staphylococcus together with methicillin-resistant strains and one vancomycin-resistant strain of the genus *Enterococcus* were kindly provided by the Department of Clinical Microbiology, University Hospital in Hradec Králové, Czech Republic. All of these strains were taxonomically classified by using biochemical tests and matrix-assisted laser desorption ionization–time of flight (MALDI-TOF) instrumentation (Microflex LH/SH MALDI-TOF, Bruker Biotyper 3.0 SW; Bruker Daltonics GmbH, Germany). The susceptibility/resistance profiles of the strains were determined by the disc diffusion method according to EUCAST recommendations (41). The vancomycin-resistant Staphylococcus aureus (VRSA) reference strain CCM 1767 was purchased from the Czech Collection of Microorganisms (CCM), and the second VRSA strain was kindly provided by the National Institute of Public Health, Prague, Czech Republic. Lists of clinical strains with susceptibility profiles and other strain specifications are presented in Table 1 and Table S4 in the supplemental material.

The broth microdilution method, according to EUCAST recommendations (with slight modifications), was employed for the evaluation of activity against these strains. An identical procedure was carried out as described in Text S1.1 in the supplemental material. To avoid genetic and epigenetic changes, which can occur due to multiple passages under *in vitro* conditions, especially in clinical isolates, we performed all experiments with only cryopreserved stock bacterial cultures.

### Categorization of clinical isolates of the genus Staphylococcus into biofilm phenotype groups.

For the purpose of categorizing biofilm production phenotypes, a quantitative test described previously by Christensen et al. (42) was used. Bacterial colonies were inoculated into tryptic soy broth medium (pH 7.3 ± 0.2) (Sigma-Aldrich, USA), and bacterial suspensions at a final density corresponding to an optical density (OD) of a 0.1 McFarland standard were prepared. Individual wells of 96-well sterile, flat-bottom, polystyrene plates (Sigma-Aldrich, USA) were filled with 150 μL of the homogeneous suspension in eight replicates. Negative-control wells containing sterile cultivation medium were included in the assays as well. The plates were incubated at 37°C in a humid atmosphere for 48 h.

After cultivation for 48 h, the biofilms that formed on the bottom and the walls of the wells were rinsed three times using a sterile 0.9% saline solution and then left to air dry. Subsequently, the biofilms were fixed with ice-cold methanol for 15 min at 4°C. Next, an aliquot of 170 μL of a freshly filtered 0.05% crystal violet solution per well was added to both plates with fixed biofilms and empty 96-well plates, where pin lids were later placed. After incubation for 20 min at room temperature, all excess stain was removed by rinsing the wells with deionized sterile water. The fixed crystal violet stain was dissolved in a mixture of ethanol-acetone (80:20, vol/vol) for 15 min at room temperature. The optical density of the dissolved crystal violet was read at 630 nm using a plate reader (Synergy HTX multimode reader; BioTek, USA).

Staphylococcal strains were classified into different categories according to the measured optical density of the eluted crystal violet (ODcv) that was bound to the produced biofilm biomass. Four categories were introduced. If the ODcv was lower than or equal to the ODc (optical density of the negative control), the strains were considered nonadherent. If the ODc was lower than the ODcv and the ODcv was lower than or equal to 2× ODc, the strains were classified as weak biofilm producers; if 2× ODc was lower than the ODcv and the ODcv was lower than or equal to 4× ODc, the strains were included in the group of moderate biofilm producers; and if 4× ODc was lower than the ODcv, the strains were considered strong biofilm producers (43).

### Determination of bacterial viability by the streak plate method.

Aliquots of 10 μL were taken at time intervals of 0, 2, 4, 6, and 24 h. The CFU per milliliter were determined by plating serially diluted samples onto Mueller-Hinton agar (MHA; Himedia, India) after incubation for 24 h at 37°C in a humid atmosphere.

### Time-kill kinetics assay.

Time-kill studies were carried out based on NCCLS guideline M26-A (44), with slight modifications. The time-kill kinetics of BH77 against MRSA ATCC 43300 (American Type Culture Collection) were tested in microtiter plates. The tested compound BH77 was dissolved in DSMO to produce a stock solution. Next, solutions of BH77 in cation-adjusted Mueller-Hinton broth (CAMHB; Sigma-Aldrich, USA) corresponding to final concentrations equal to the MIC, 2× MIC, and 0.25× MIC (for viability evaluation using the streak plate method) and the MIC, 2× MIC, 0.5× MIC, 0.25× MIC, and 0.125× MIC (for evaluation of metabolic activity) were prepared and transferred to wells of microtiter plates in quadruplicates (for the streak plate method) and triplicates (for metabolic activity evaluation). The final concentration of DMSO in solutions and the included controls corresponded to 1% (vol/vol). The initial bacterial inoculum in CAMHB corresponding to approximately 5 × 10^5^ CFU/mL was added to the wells with BH77 solutions and incubated at 37°C in a humid atmosphere.

### Determination of bacterial metabolic activity by an MTT colorimetric assay.

A concentration of 5 mg/mL MTT [3-(4,5-dimethylthiazol-2-yl)-2,5-diphenyltetrazolium bromide] (Sigma-Aldrich, USA) was prepared in phosphate-buffered saline (PBS) (pH 7.2). At time intervals of 0, 2, 4, 6, and 24 h of incubation, 12 μL of the MTT solution was added to every well, and 96-well microtiter plates were incubated for 2.5 h at 37°C in a humid atmosphere. After incubation with the MTT solution, MTT lysis buffer (100 μL of a 10% sodium dodecyl sulfate solution in 0.01 M HCl) was added, and the OD was measured at 540 nm. The ultimate OD for each well was calculated as the OD (at 540 nm) of the sample minus the OD (at 540 nm) of the control (culture medium and test compound solvent).

### Checkerboard assay.

A screening test for potential synergy was performed using a broth microdilution checkerboard method. The assays were performed in honeycomb plates (Oy Growth Curves, Finland) in a 10- by 10-well configuration. Four antibiotic agents used for the treatment of infections caused by Gram-positive bacteria, VAN, SXT, RIF, and CIP, were selected for the combinations (compounds A+B) BH77+VAN, BH77+SXT, BH77+RIF, and BH77+CIP. MRSA ATCC 43300 was used as the bacterium for performing the MIC evaluation and the checkerboard assay.

Briefly, the compounds were dissolved in DMSO (Sigma-Aldrich, USA) to produce stock solutions. The final concentration of DMSO in the testing medium corresponded to 1% (vol/vol) of the total solution composition and did not affect the growth of the bacteria. Solutions of BH77 and selected antibiotic drugs were prepared separately. Finally, solutions with different concentrations of the tested compound and antibiotic drugs were transferred to a honeycomb plate at a volume ratio of 1:1. The first compound was transferred in a horizontal direction, and the second one was transferred in a vertical direction. Each well with a final volume of 200 μL of the compound mixture was inoculated with the MRSA bacterial strain (the final density corresponded to 5 × 10^5^ CFU) in CAMHB. Positive controls (bacteria in CAMHB and 1% [vol/vol] DMSO), negative controls (CAMHB and 1% [vol/vol] DMSO), and compound A (or B) alone were included in each assay as well. The honeycomb plates were incubated in a Bioscreen C instrument (Oy Growth Curves, Finland) at 36.8°C for 20 h. At each 30 min of incubation, the OD was recorded at 580 nm. After incubation, the percent inhibition in each well was compared to those of the positive controls (the background values were subtracted). Visual inspection and the metabolic activity indicator alamarBlue (alamarBlue cell viability reagent; Thermo Fisher Scientific, USA) were used for evaluating MIC endpoints.

The total fractional inhibitory concentration (FIC) index (ΣFICI) was used to interpret the results of the checkerboard assays. The FIC index was calculated according to the following formula: ΣFICI = FIC(A) + FIC(B). FIC(A) and FIC(B) were calculated as follows: FIC(A or B) = MIC of drug A (or B) in combination/MIC of drug A (or B) alone. The FIC index results were defined as follows: a ΣFICI of ≤0.5 indicates synergy, 0.5 < ΣFICI ≤ 1 indicates additivity, 1 < ΣFICI ≤ 4 indicates indifference, and a ΣFICI of >4 indicates antagonism.

### Screening of *in vitro* antibiofilm activity.

An assay to screen for *in vitro* antibiofilm activity was carried out using the biofilm microtiter plate method, and two biofilm-forming strains, MRSA ATCC 43300 and Staphylococcus epidermidis ATCC 1228 (CCM 784), were used to evaluate the MBIC (minimum biofilm inhibition concentration) and MBEC (minimum biofilm eradication concentration).

Briefly, inocula were prepared from bacteria cultivated on MHA plates for 18 h at 37°C in a humid atmosphere. The density of the bacterial suspension was adjusted to 5 × 10^5^ CFU/mL in tryptic soy broth (Himedia, India), and final volumes of 100 μL were seeded into flat-bottom, polystyrene, non-tissue-culture-treated microtiter plates. A negative control (medium only without any bacterial agents) was also included. The plates were then incubated in a humidified incubator for 24 h. After biofilm formation, the wells were washed three times with a sterile 0.9% saline solution. The plates were left to air dry for 15 min.

A stock solution of the tested compound BH77 was prepared in DMSO. Subsequently, solutions of BH77 in CAMHB at concentrations ranging from 3.125 mg/mL to 0.0244 mg/mL with a final concentration of DMSO of 1% (vol/vol) were prepared. CIP (Sigma-Aldrich, USA) was used as a reference antibiotic in the assay. A stock solution of CIP was prepared analogously, in DMSO, and solutions of CIP in CAMHB (1% [vol/vol] DMSO) corresponding to concentrations ranging from 0.0244 mg/mL to 0.000381 mg/mL were subsequently prepared. Solutions with various compound concentrations were transferred to wells with preformed bacterial biofilms in triplicate in a total volume of 100 μL. The biofilms were exposed to drugs for 24 h at 37°C without any shaking. After 24 h of incubation, 90 μL of the supernatant was transferred to the external plates for the purpose of determining the MBIC value. Ten microliters of the metabolic indicator alamarBlue (alamarBlue cell viability reagent; Thermo Fisher Scientific, USA) was transferred to each well of the external microtiter plates, and the plates were incubated for 30 min at 37°C in gentle shaking mode. After incubation, visual inspection and fluorescence measurements (excitation wavelength [λEx] of 530 nm and emission wavelength [λEm] of 590 nm) (Synergy HTX multimode microplate reader; BioTek, Winooski, VT, USA) were performed. The MBIC value was defined as the minimum antimicrobial concentration at which there was no evidence of observable metabolic activity associated with bacterial growth in planktonic bacteria forming a biofilm.

The wells of the microtiter plate with the preformed staphylococcal biofilm challenged by the tested antimicrobial agent were washed four times with a sterile 0.9% saline solution. After washing, 20 μL of CAMHB medium was transferred to each well of the plate. Next, to release the attached biofilm-forming bacteria into the CAMHB medium, two sonication steps (5 min, with a vigorous shaking step between the two sonication procedures) were performed. To evaluate the MBEC, the spot plate count method was used. The released biofilm-forming bacteria with a total volume of 10 μL from each well were seeded onto either Baird-Parker agar (Himedia, India) or MHA plates and cultivated for 24 h at 37°C. The MBEC value was defined as the minimum antimicrobial concentration at which no observable bacterial growth or a limited number of CFU (limit of 50 CFU per spot, corresponding to a 99.9% loss of viability compared to the initial biofilm inoculum) was detected. Each experiment was repeated twice in triplicate.

### Evaluation of *in vivo* toxicity using the Galleria mellonella animal model.

Galleria mellonella animals were reared in the laboratory of the Department of Biological and Medical Sciences, Faculty of Pharmacy in Hradec Králové, Charles University. The larvae were fed an artificial diet described previously by Haydak (45) and reared in the dark at 30°C. Only fully vital, cream-colored larvae with a weight ranging from 250 to 300 mg were selected for test compound administration before each experiment. In brief, the test compound BH77 was dissolved in DMSO and diluted to the required working concentration with PBS (pH 7.4) (Sigma-Aldrich, USA). The final concentration of DMSO corresponded to 30% (vol/vol). Ten microliters of the sample per larva was administered to the larvae in two different ways: into the hemocoel through the last left proleg and by the feeding route (force-feeding method) using a Hamilton syringe. For each experiment, two control groups (an untreated control and a control administered 30% [vol/vol] DMSO in PBS) were also included. The inoculated larvae and control groups were then incubated in petri dishes at 37°C, and mortality was monitored at 1-, 24-, 72-, and 120-h intervals. Death was defined as the complete loss of mobility, including a physical stimulus using a plastic pipette. At first, basic screening survival experiments with limited numbers of individuals (with regard to ethical compliance) were included in this study: 70 individuals for intrahemocoel administration (groups 1 to 6, *n *= 9; control groups, *n *= 8) and 48 individuals for peroral administration (all groups, *n *= 6). Subsequently, to obtain more relevant data outputs, selected groups with higher administered doses were reinforced by additional individuals (hemocoel administration, *n *= 28; peroral administration, *n *= 49). The inoculated larvae and control groups were then incubated in petri dishes at 37°C. The survival of the larvae was recorded over a 120-h period (1, 24, 72, and 120 h after administration). Death was defined as the complete loss of mobility, including a physical stimulus using a plastic pipette. The mortality for each dose/time interval was calculated, and survival curves were designed via software analysis (GraphPad Prism 9.0.0).

### Statistical analysis.

Survival experiments in the *in vivo* toxicity studies included 219 individuals. A total of 138 individuals were included for the evaluation of the *in vivo* toxicity of BH77 after intrahemocoel administration (group 1, *n *= 21; groups 2 and 3, *n *= 23; groups 4 to 6, *n *= 9; control groups, *n *= 24 and *n *= 20), and 81 individuals were included for the evaluation of toxicity after peroral administration (group 1, *n *= 15; groups 2 and 3, *n *= 16; groups 4 to 6, *n *= 6; control groups, *n *= 16). All data outputs were analyzed by using GraphPad Prism software version 9.0.0. Data from the survival experiments were subjected to the log rank Mantel-Cox (curve comparison) test and the Mantel-Haenszel (hazard ratio) pairwise comparison test. The results were considered significant at a *P* value of <0.05 for all analyses.
