# Supplementary material for: Insight into the Antibacterial Action of Iodinated Imine, an Analogue of Rafoxanide: a Comprehensive Study of Its Antistaphylococcal Activity
Source: Microbiol Spectr. 2023 Apr 26;11(3):e03064-22. doi: 10.1128/spectrum.03064-22 (PMC10269765; doi:10.1128/spectrum.03064-22)
Supplement: Supplemental file 1 — Tables S1 to S9, Fig. S1 to S4, and supplemental text. Download spectrum.03064-22-s0001.pdf, PDF file, 0.6 MB [file spectrum.03064-22-s0001.pdf]

Insight into the antibacterial action of iodinated imine, analogue of rafoxanide – a comprehensive study of the antistaphylococcal activity

Martin Krátký[a,#], Klára Konečná[b,#], Ondřej Janďourek[b], Adéla Diepoltová[b], Pavlína Vávrová, Barbora Voxová[b], Marcela Vejsová[b], Pavel Bárta[c], Szilvia Bősze[d]

[a]Charles University, Faculty of Pharmacy in Hradec Králové, Department of Organic and Bioorganic Chemistry, Hradec Králové, Czech Republic

[b]Charles University, Faculty of Pharmacy in Hradec Králové, Department of Biological and Medical Sciences, Hradec Králové, Czech Republic

[c]Charles University, Faculty of Pharmacy in Hradec Králové, Department of Biophysics and Physical Chemistry, Hradec Králové, Czech Republic

[d]ELKH-ELTE Research Group of Peptide Chemistry, Budapest, Hungary

[#]Address correspondence to Martin Krátký, martin.kratky@faf.cuni.cz

Charles University, Faculty of Pharmacy in Hradec Králové, Department of Organic and Bioorganic Chemistry, Czech Republic, Tel: + (420) 495067302, Fax: + (420) 495067166, ORCID: 0000-0002-4600-8409

[#]Address correspondence to Klára Konečná, konecna@faf.cuni.cz

Charles University, Faculty of Pharmacy in Hradec Králové, Department of Biological and Medical Sciences, Czech Republic, Tel: + (420)495067366, Fax: + (420)495518002, ORCID:0000-0001-5670-7767

SUPPLEMENTARY MATERIALS

Supplemental file 1: **Results – Table S1** Structural and physicochemical parameters of BH77 for prediction of drug-likeness; **Table S2** Antibacterial activity of BH77 against reference bacterial strains; **Table S3** Evaluation of the susceptibility of reference bacterial strains to conventional antibiotics, ciprofloxacin, gentamicin, and vancomycin; **Table S4** List of bacterial strains, their susceptibility/resistance profiles, used for advanced screening of antibacterial activity; **Table S5** Reduction of methicillin-resistant *Staphylococcus aureus* strain (ATCC 433000) viability after 24-hours exposure to different concentrations of BH77; **Table S6** Total fractional inhibitory concentration index FICI (∑FICI) determined by checkerboard assay of vancomycin (VAN) and BH77, trimethoprim-sulfamethoxazole (SXT) and BH77, ciprofloxacin (CIP) and BH77, rifampicin (RIF) and BH77 combinations with methicillin-resistant *Staphylococcus aureus* (ATCC 43300); **Table S7** Evaluation of *in vivo* toxicity of BH77 in animal model, larvae of *Galleria mellonella*; **Table S8** Survival analyses of *Galleria mellonella* larvae after intra hemocoel administration of BH77; **Table S9** Survival analyses of *Galleria mellonella* larvae after peroral administration of BH77; **Fig. S1** Pharmacodynamic study of *in vitro* interaction of BH77 and three *Staphylococcus aureus* strains; **Fig. S2** Results of macromolecular assay; **Fig. S3** $DiSC_3(5)$-based kinetic fluorescence measurement for detection of the impact of BH77 on membrane depolarization in *Staphylococcus aureus*; **Fig. S4** *In vitro* cytotoxic effect of the tested compound, BH77.

Methods – S1.1 Basic screening of antibacterial activity; **S1.2** *In silico* prediction of physicochemical and structural parameters; **S1.3** Determination of bacteriostatic vs. bactericidal activity; **S1.4** Evaluation of *in vitro* pharmacodynamic interaction; **S1.5** Determination of mechanism of action by macromolecular biosynthesis assay; **S1.6 Determination of the impact of BH77 on staphylococcal cytoplasmic membranes; S1.7** Evaluation of BH77 *in vitro* cytotoxicity.

Table S1 Structural and physicochemical parameters of BH77 for prediction of drug-likeness

Parameter	Value	Fitting criteria
**MW**	497.50	✔
**log*P***	4.75	✔
**H-bond donors**	1	✔
**H-bond acceptors**	2	✔
**GI absorption**	High	✔
**Pan Assay Interference Structure**	No	✔

Table S2 Antibacterial activity of BH77 against reference bacterial strains. Antibacterial action of the BH77 compound was evaluated by the microdilution method according to EUCAST recommendations, with slight modifications. Minimum inhibitory concentration (MIC) of BH77 was evaluated after 24 and 48 hours of cultivation by visual inspection and spectrophotometric measurement.

	**MIC (mg/l) / (μM)**	
Cultivation time (hours)	**24 h**	**48 h**
**Gram-positive bacterial strains**		
SA (CCM 4223, ATCC 29213)	7.773-15.549 / 15.625-31.25	15.549 / 31.25
MRSA (CCM 4750, ATCC 43300)	7.773-31.094 / 15.625-62.5	31.094 / 62.5
SE (CCM 4418, ATCC 12228)	15.549-31.094 / 31.25-62.5	31.094 / 62.5
EF (CCM 4224, ATCC 29212)	31.094-62.188 / 62.5-125.0	31.094-62.188 / 62.5-125
**Gram-negative bacterial strains**		
EC (ATCC 25922, CCM 3954)	>248.736 / >500	>248.736 / >500
KP (ATCC 10031, CCM 4415)	>248.736 / >500	>248.736 / >500
AB (ATCC 19606, DSM 30007)	>248.736 / >500	>248.736 / >500
PA (ATCC 27853, CCM 3955)	>248.736 / >500	>248.736 / >500

Footnote: CCM – Czech Collection of Microorganisms, ATCC – American Type Culture Collection, DSM – German Collection of Microorganisms and Cell Cultures; SA – *Staphylococcus aureus* subsp. *aureus*, MRSA – methicillin-resistant *Staphylococcus aureus* subsp. *aureus*, SE – *Staphylococcus epidermidis*, EF – *Enterococcus faecalis*, EC – *Escherichia coli*, KP – *Klebsiella pneumoniae*, AB – *Acinetobacter baumannii*, PA – *Pseudomonas aeruginosa*

Table S3 Evaluation of the susceptibility of reference bacterial strains to conventional antibiotics, ciprofloxacin, gentamicin, and vancomycin. The bacterial strain susceptibility was evaluated by the microdilution method according to EUCAST recommendations, with slight modifications. Minimum inhibitory concentration was evaluated after 24 hours by visual inspection and spectrophotometric measurement.

Internal quality standard	Ciprofloxacin				Gentamicin sulphate				Vancomycin hydrochloride			
Bacterial strain	* (mg/l)	* (μM)	** (mg/l)	** (μM)	* (mg/l)	* (μM)	** (mg/l)	** (μM)	* (mg/l)	* (μM)	** (mg/l)	** (μM)
SA	0.256	0.773	0.256	0.773	1.0	1.936	0.5	0.968	2.0	1.3461	2.0	1.346
MRSA	0.128	0.386	0.128	0.386	>8.0	>15.486	>8.0	>15.486	1.0	0.673	2.0	1.346
SE	0.256	0.773	0.128	0.386	0.125	0.242	0.125	0.242	4.0	2.692	4.0	2.692
EF	0.512	1.545	1.024	3.090	>8.0	>15.486	>8.0	>15.486	4.0	2.692	4.0	2.692
EC	0.008	0.024	0.008	0.024	1.0	1.936	1.0	1.936	>32	>21.536	>32	>21.536
KP	0.008	0.024	0.008	0.024	0.5	0.968	0.5	0.968	>32	>21.536	>32	>21.536
AB	0.512	1.545	0.256	0.773	8.0	15.486	8.0	15.486	>32	>21.536	>32	>21.536
PA	0.512	1.545	0.512	1.545	0.5	0.968	0.5	0.968	>32	>21.536	>32	>21.536

Footnote: SA – *Staphylococcus aureus* subsp. *aureus* ATCC 29213, MRSA – *Staphylococcus aureus* subsp. *aureus* MRSA, ATCC 43300, SE – *Staphylococcus epidermidis*, ATCC 12228, EF – *Enterococcus faecalis* ATCC 29212, EC – *Escherichia coli* ATCC 25922, KP – *Klebsiella pneumoniae* ATCC 10031, AB – *Acinetobacter baumannii* ATCC 19606, PA – *Pseudomonas aeruginosa* ATCC 27853, ATCC – American Type Culture Collection

*Spectrophotometric detection – results were read with a microdilution plate reader (SynergyTM HTX. BioTek Instruments. Inc., USA) at wavelength 530 nm. The MIC of antibacterial agents is the lowest concentration giving rise to an inhibition of growth of 95% of that of the drug-free control. Results were read after 24 h microdilution plates cultivation without agitation at 35±2°C in humidified atmosphere.

**The MIC was determined by naked eye in the well with the lowest drug concentration where no visible growth of microbial agent was detected. Results were read after 24 h.

Table S4 List of bacterial strains, their susceptibility/resistance profiles, used for advanced screening of antibacterial activity

ID No. (strain specification)	Bacterial strain	Susceptibility (S)/resistance (R) profile*
131/16	methicillin-resistant *Staphylococcus aureus*	S: nitrofurantoin, trimethoprim-sulfamethoxazole, tetracycline<br>R: cefoxitin, clindamycin, erythromycin
136/16	*Staphylococcus aureus*	S: cefoxitin, clindamycin, nitrofurantoin, trimethoprim-sulfamethoxazole, tetracycline<br>R: erythromycin
138/16	methicillin-resistant *Staphylococcus aureus*	S: clindamycin, nitrofurantoin, trimethoprim-sulfamethoxazole, tetracycline<br>R: cefoxitin, erythromycin
141/16	*Staphylococcus aureus*	S: cefoxitin, clindamycin, erythromycin, nitrofurantoin, trimethoprim-sulfamethoxazole, tetracycline
143/16 (ATCC 43300, CCM 4750)	methicillin-resistant *Staphylococcus aureus*	******S: nitrofurantoin, trimethoprim-sulfamethoxazole, tetracycline, linezolid<br>******R: clindamycin, erythromycin
153/16	methicillin-resistant *Staphylococcus aureus*	S: gentamicin, linezolid, rifampicin, tigecycline, vancomycin<br>R: ciprofloxacin
154/16	*Staphylococcus aureus*	S: nitrofurantoin, tetracycline, trimethoprim-sulfamethoxazole<br>R: cefoxitin, clindamycin, erythromycin
198/16	vancomycin-resistant *Enterococcus faecium*	S: linezolid<br>R: ampicillin, gentamicin, teicoplanin, tigecycline, vancomycin
205/18 (ATCC 12228, CCM 7844)	*Staphylococcus epidermidis*	******S: oxacillin, gentamicin, erythromycin, clindamycin, linezolid<br>******R: tetracycline
203/19 (NIPH)	vancomycin-resistant *Staphylococcus aureus*	ND

206/19 (CCM 1767)	vancomycin-resistant *Staphylococcus aureus*	ND

ID No. – internal laboratory identification number, ND – not determined, , ATCC – American Type Culture Collection, USA, CCM – Czech Collection of Microorganisms, Czech Republic, NIPH – The National Institute of Public Health in Prague, Czech Republic, * disc diffusion test according to EUCAST recommendation, **evaluation of the susceptibility/resistance profile by microdilution broth method, according to EUCAST recommendation**,** susceptibility/resistance profile taken from German collection of microorganisms (DSM), and the associated database, *BacDive*, BacDive | The Bacterial Diversity Metadatabase (dsmz.de)

Table S5 Reduction of methicillin-resistant *Staphylococcus aureus* strain (ATCC 433000) viability after 24-hours exposure to different concentrations of BH77. The microdilution broth method and spread plate technique for colony forming units calculation were employed for evaluation.

$c_{(BH77)}$, µM	×-fold of MIC	% of reduction*
31.25	1×MIC	99.89
62.5	2×MIC	99.91
125	4×MIC	99.92
250	8×MIC	99.93

Footnote: MIC – minimum inhibitory concentration, * reduction in the number of colony-forming unit (CFU) compared to CFU number of initial bacterial inoculum

Table S6 Total fractional inhibitory concentration index FICI (∑FICI) determined by checkerboard assay of vancomycin (VAN) and BH77, trimethoprim-sulfamethoxazole (SXT) and BH77, ciprofloxacin (CIP) and BH77, rifampicin (RIF) and BH77 combinations with methicillin-resistant *Staphylococcus aureus* (ATCC 43300). The minimum inhibitory concentration (MIC) of compounds alone corresponds to: $MIC_{(VAN)}$ = 1 mg/l, $MIC_{(BH77)}$ = 31.25 μM in VAN+BH77 combination; $MIC_{(SXT)}$ = 0.5 mg/l and $MIC_{(BH77)}$= 15.625 μM in SXT+BH77 combination; $MIC_{(CIP)}$ = 0.5 mg/l and $MIC_{(BH77)}$ = 31.25 μM in CIP+BH77 combination and $MIC_{(RIF)}$ = 0.0025 mg/l and $MIC_{(BH77)}$ = 15.625 μM in RIF+BH77 combination.

Combination of compounds (vancomycin : BH77)			
MIC (VAN : BH77) μM	MIC (VAN : BH77) mg/l	∑FICI	Interpretation
0.006 : 62.5	0.008 : 31.094	2.008	NA
0.011 : 62.5	0.016 : 31.094	2.016	NA
0.021 : 62.5	0.031 : 31.094	2.031	NA
0.042 : 62.5	0.063 : 31.094	2.063	NA
0.084 : 62.5	0.125 : 31.094	2.125	NA
0.168 : 62.5	0.25 : 31.094	1.25	NA
0.337 : 31.25	0.5 : 15.546	1.5	NA
0.673 : 15.625	1.0 : 7.773	1.5	NA
1.346 : 7.813	2.0 : 3.887	2.250	NA
1.346 : 3.906	2.0 : 1.943	2.125	NA
1.346 : 0.977	2.0 : 0.4858	2.032	NA
**Combination of compounds (trimethoprim-sulfamethoxazole : BH77)**			
MIC (SXT : BH77) μM	MIC (SXT : BH77) mg/l	∑FICI	Interpretation
0.058 : 31.25	0.032 : 15.554	2.063	NA
0.230 : 62.5	0.125 : 31.108	4.25	AN
0.460 : 31.25	0.25 : 15.554	2.5	NA
0.920 : 31.25	0.5 : 15.554	3.0	NA
1.840 : 31.25	1.0 : 15.554	4.0	NA
3.679 : 15.625	2.0 : 7.777	5.0	AN
3.679 : 7.813	2.0 : 3.888	4.5	AN
3.679 : 3.906	2.0 : 1.944	4.25	AN
3.679 : 1.953	2.0 : 0.972	4.125	AN

3.679 : 0.977	2.0 : 0.486	4.063	AN
Combination of compounds (ciprofloxacin : BH77)			
MIC (CIP : BH77) µM	MIC (CIP : BH77) mg/l	∑FICI	Interpretation
0,003 : 62.5	0.002 : 31.108	2.004	NA
0.006 : 62.5	0.004 : 31.108	2.008	NA
0.012 : 62.5	0.008 : 31.108	2.016	NA
0.024 : 62.5	0.016 : 31.108	2.031	NA
0.047 : 62.5	0.032 : 31.108	2.063	NA
0.094 : 62.5	0.063 : 31.108	2.125	NA
0.189 : 31.25	0.125 : 15.553	1.25	NA
0.377 : 15.625	0.25 : 7.777	1	NA
0.377 : 7.813	0.25 : 3.888	0.75	NA
0.377 : 3.906	0.25 : 1.944	0.625	NA
0.755 : 1.953	0.5 : 0.972	1.062	NA
0.755 : 0.977	0.5 : 0.486	1.031	NA
Combination of compounds (rifampicin : BH77)			
MIC (RIF×10^{-3} : BH77) µM	MIC (RIF×10^{-3} : BH77) mg/l	∑FICI	Interpretation
0.19 : 62.5	0.156 : 31.108	4.062	AN
0.76 : 62.5	0.625 : 31.108	4.25	AN
1.52 : 62.5	1.25 : 31.108	4.5	AN
3.04 : 62.5	2.5 : 31.108	5.0	AN
6.08 : 31.25	5.0 : 15.553	4.0	NA
6.08 : 15.625	5.0 : 7.777	3.0	NA
6.08 : 7.813	5.0 : 3.888	2.5	NA
6.08 : 3.906	5.0 : 1.944	2.25	NA
6.08 : 1.953	5.0 : 0.972	2.125	NA
6.08 : 0.977	5.0 : 0.486	2.063	NA

Footnote: NA – non-antagonistic effect, AN – antagonistic effect

Table S7 Evaluation of *in vivo* toxicity of BH77 in animal model, larvae of *Galleria mellonella*. Two different ways of compound administration were included, into hemocoel (A), and per oral (B).

A.

		Group one		Group two		Group three		Group four		Group five		Group six		Control group		Control group	
	Dose (mg/kg of body weight) / Hours (visual inspection after administration)	1600.0-2000.0	(%)*	400.0-500.0	(%)*	240.0-300.0	(%)*	120.0-150.0	(%)*	40.0-50.0	(%)*	4.0-5.0	(%)*	10 ml of PBS + 30% (v/v) DMSO	(%)*	w/o any administration	(%)*
Number of surviving larvae/mortality (%)	1	21	0%	23	0	23	0	9	0	9	0	9	0	24	0	20	0
	24	17	19.05	23	0	22	4.35	9	0	8	11.11	9	0	23	4.17	20	0
	72	17	19.05	23	0	21	8.7	9	0	7	22.22	8	11.11	23	4.17	20	0
	120	15	28.57	23	0	20	13.04	9	0	7	22.22	8	11.11	23	4.17	20	0

B.

		Group one		Group two		Group three		Group four		Group five		Group six		Control group		Control group	
	Dose (mg/kg of body weight) / Hours (visual inspection after administration)	1600.0-2000.0	(%)*	400.0-500.0	(%)*	240.0-300.0	(%)*	120.0-150.0	(%)*	40.0-50.0	(%)*	4.0-5.0	(%)*	10 ml of PBS + 30% (v/v) DMSO	(%)*	w/o any administration	(%)*
Number of surviving larvae/mortality (%)	1	15	0	16	0	16	0	6	0	6	0	6	0	16	0	16	0
	24	13	13.33	14	12.5	15	6.25	6	0	6	0	5	16.67	16	0	16	0
	72	8	46.67	14	12.5	15	6.25	6	0	6	0	5	16.67	16	0	16	0
	120	8	46.67	14	12.5	15	6.25	6	0	6	0	5	16.67	16	0	16	0

Footnote: (%)* – mortality mediated by BH77 in %, PBS – phosphate saline buffer, w/o administration – without administration, DMSO – dimethyl sulfoxide

Table S8 Survival analyses of *Galleria mellonella* larvae after intra hemocoel administration of BH77. Values represent outputs from pairwise comparison Log-rank Mantel-Cox test and Hazard Ratio(s) (Mantel-Haenszel) test with CI 95%.

BH77 (mg/kg of body weight)			
	1600-2000 (B)	400-500 (C)	240-300 (D)
0 (control group – 30% DMSO +PBS (A)	Significance: * $\chi^{2\,(1)} = 4.935$ P = 0.0263 <br>A/B = 0.1734 (0.03696 to 0.8136) B/A = 5.767 (1.229 to 27.06)	Significance: **ns** $\chi^{2\,(1)} = 0.9583$ P = 0.3276 <br>A/C = 7.088 (0.1405 to 357.5) C/A = 0.1411 (0.002797 to 7.117)	Significance: **ns** $\chi^{2\,(1)} = 1.126$ P = 0.2885 <br>A/D = 0.3438 (0.04787 to 2.469) D/A = 2.909 (0.4050 to 20.89)
1600-2000 (B)		Significance: ** $\chi^{2\,(1)} = 7.478$ P = 0.0062 <br>B/C = 0.09951 (0.01904 to 0.5201) C/B =10.05 (1.923 to 52.53)	Significance: **ns** $\chi^{2\,(1)} = 1.621$ P = 0.2030 <br>B/D = 0.4135 (0.1062 to 1.610) D/B = 2.418 (0.6210 to 9.416)
400-500 (C)			Significance: **ns** $\chi^{2\,(1)} = 3.140$ P = 0.0764 <br>C/D = 0.1291 (0.01342 to 1.243) D/C = 7.744 (0.8047 to 74.52)

ns – no significant difference

Table S9 Survival analyses of *Galleria mellonella* larvae after per oral administration of BH77. Values represent outputs from pairwise comparison Log-rank Mantel-Cox test and Hazard Ratio(s) (Mantel-Haenszel) test with CI 95%.

BH77 (mg/kg of body weight)			
	1600-2000 (B)	400-500 (C)	240-300 (D)
0 (control group – 30% DMSO +PBS (A)	Significance: ** $\chi^{2\,(1)}$ = 9.315 P = 0.0023 <br>A/B = 0.08568 (0.01768 to 0.4151) B/A = 11.67 (2.409 to 56.55)	Significance: **ns** $\chi^{2\,(1)}$ = 2.067 P = 0.1506 <br>A/C = 0.1266 (0.007564 to 2.119) C/A = 7.898 (0.4719 to 132.2)	Significance: **ns** $\chi^{2\,(1)}$ = 1.000 P = 0.3173 <br>A/D = 0.1353 (0.002685 to 6.821) D/A = 7.389 (0.1466 to 372.4)
1600-2000 (B)		Significance: **ns** $\chi^{2\,(1)}$ = 3.611 P = 0.0574 <br>B/C = 0.2569 (0.06325 to 1.044) C/B = 3.892 (0.9581 to 15.81)	Significance: * $\chi^{2\,(1)}$ = 5.921 P = 0.0150 <br>B/D = 0.1596 (0.03641 to 0.6998) D/B = 6.265 (1.429 to 27.46)
400-500 (C)			Significance: **ns** $\chi^{2\,(1)}$ = 0.3563 P = 0.5506 <br>C/D = 0.4903 (0.04724 to 5.090) D/C = 2.039 (0.1965 to 21.17)

ns – no significant difference

A. *In vitro* pharmacodynamic study of the interaction BH77 with methicillin-resistant *Staphylococcus aureus* ATCC 43300

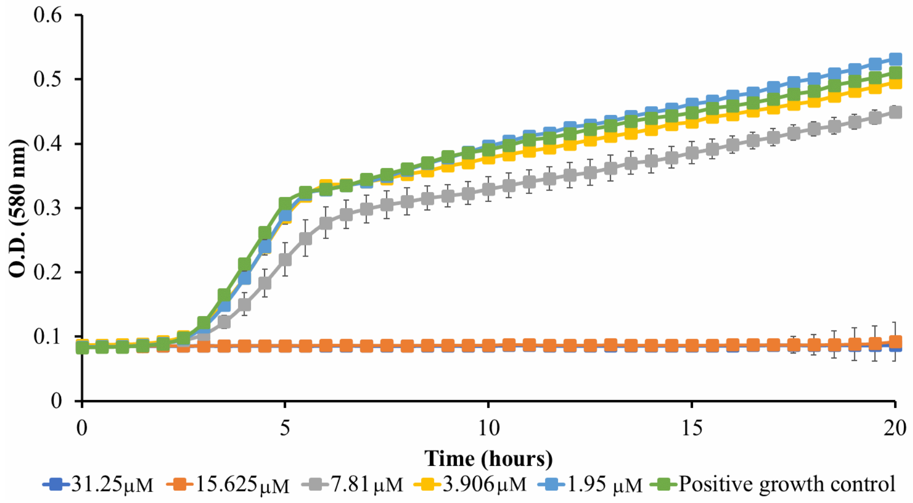

B. *In vitro* pharmacodynamic study of the interaction BH77 with vancomycin-resistant *Staphylococcus aureus* CCM 1767

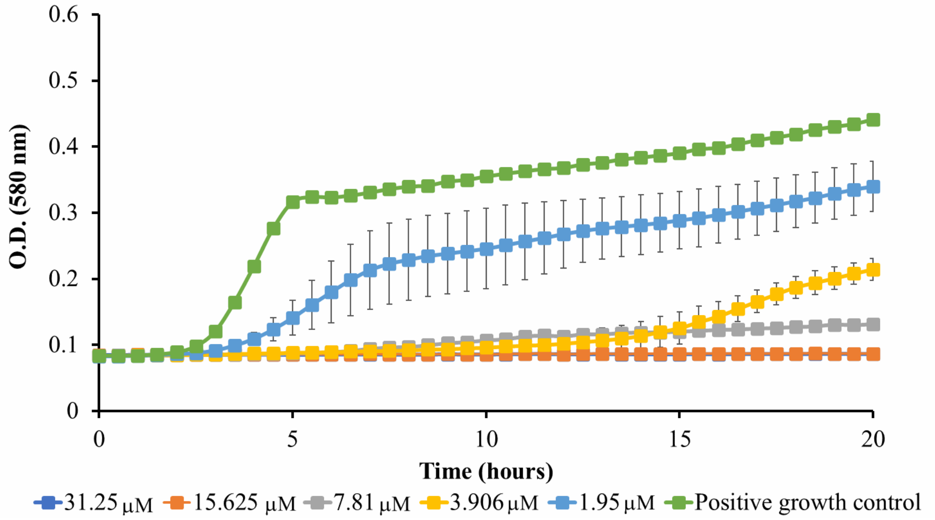

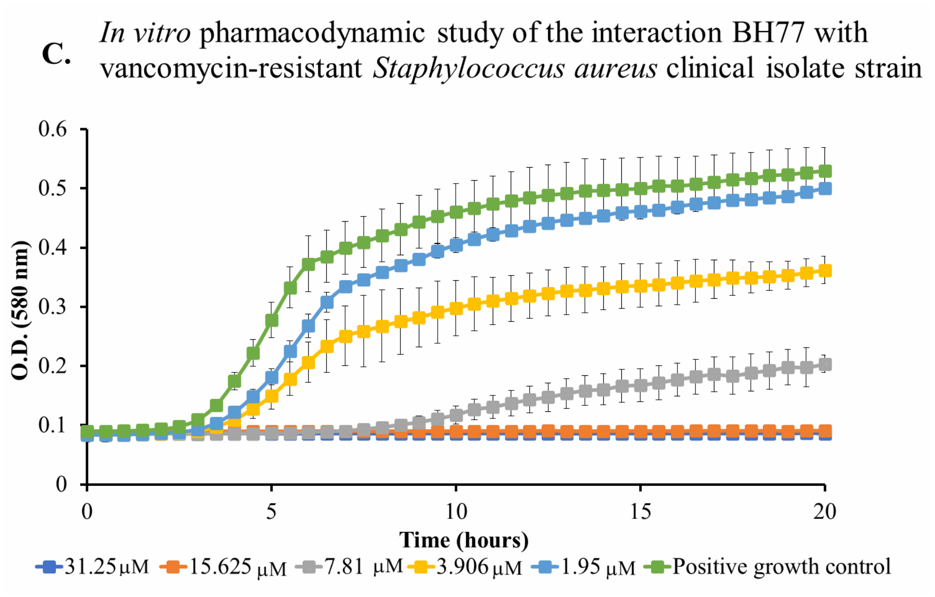

Fig. S1 Pharmacodynamic study of *in vitro* interaction of BH77 and three *Staphylococcus aureus* strains, A) reference strain methicillin-resistant *S. aureus* ATCC 43300, B) reference strain vancomycin-resistant *S. aureus* CCM 1767, and C) clinical isolate strain, vancomycin-resistant *S. aureus*. The microdilution broth method was employed for mapping the bacterial growth affected by various concentrations of BH77.

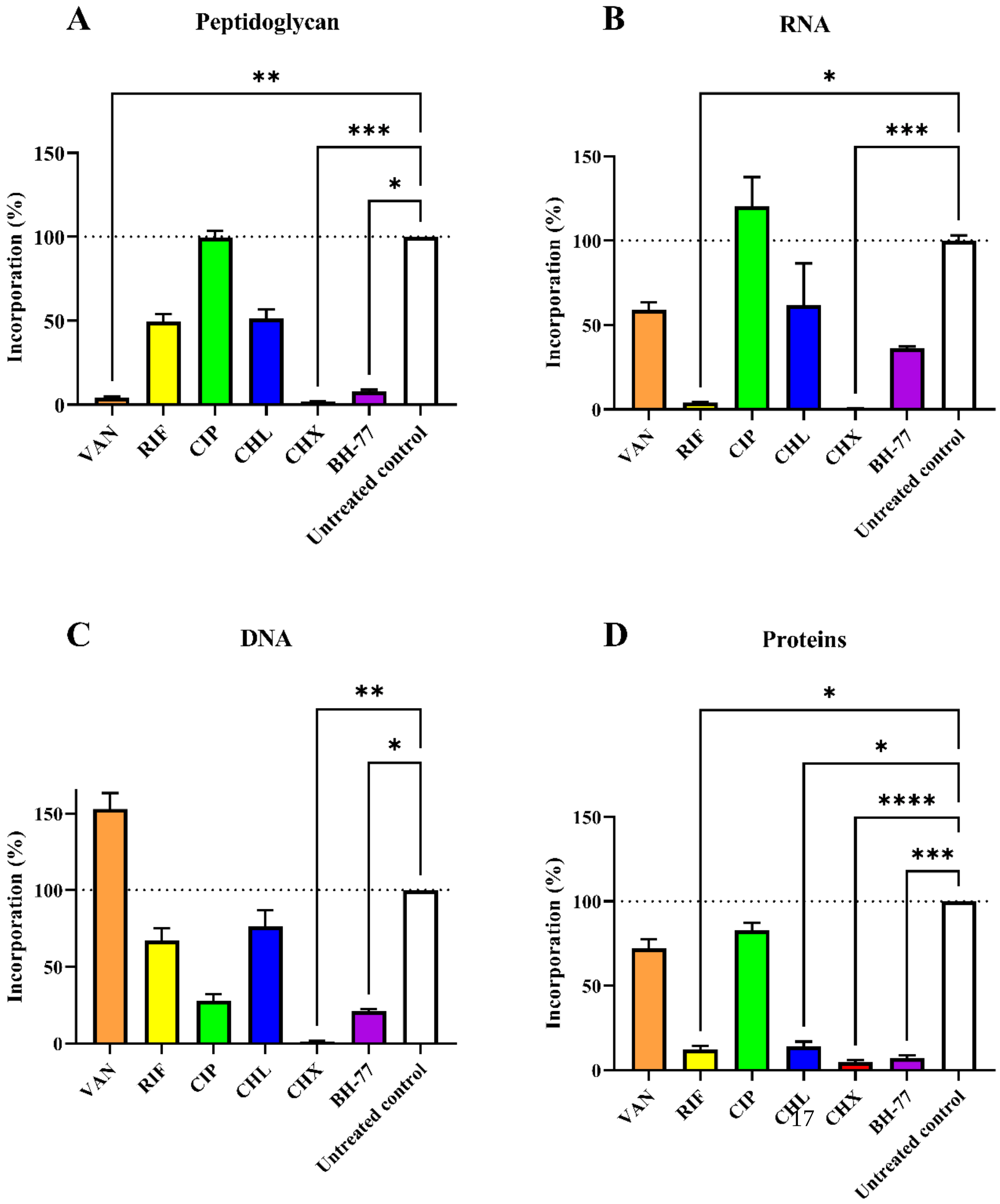
A
Peptidoglycan
B
RNA
C
DNA
D
Proteins
Incorporation (%)
0
50
100
150
VAN
RIF
CIP
CHL
CHX
BH-77
Untreated control
*
**
***
****

Fig. S2 Results of macromolecular assay – BH77 inhibits the synthesis of proteins, peptidoglycan, and DNA. Inhibition of biosynthetic pathway is indicated by lower incorporation of radioactively labelled precursors [^{3}H] *N*-acetylglucosamine (A), [^{3}H]uridine (B), [^{3}H]thymidine (C), and [^{3}H]leucine (D) by methicillin-resistant *Staphylococcus aureus* (ATCC 43300) strain treated for 2 hours at 4×MIC of vancomycin (VAN), rifampicin (RIF), ciprofloxacin (CIP), chloramphenicol (CHL), chlorhexidine (CHX), or BH-77. Results are expressed as the percentage of untreated controls. The values shown are means of three independent experiments prepared in duplicates ± SEMs. Dotted lines represent 100% incorporation of labelled macromolecules (untreated control). Significant reduction in biosynthetic pathway compared to untreated control is indicated by p-value where $p < 0.05$ was accepted as statistically significant (* $p<0.05$; ** $p<0.01$; *** $p<0.001$; **** $p<0.0001$) determined by nonparametric one-way ANOVA test (Kruskal-Wallis test).

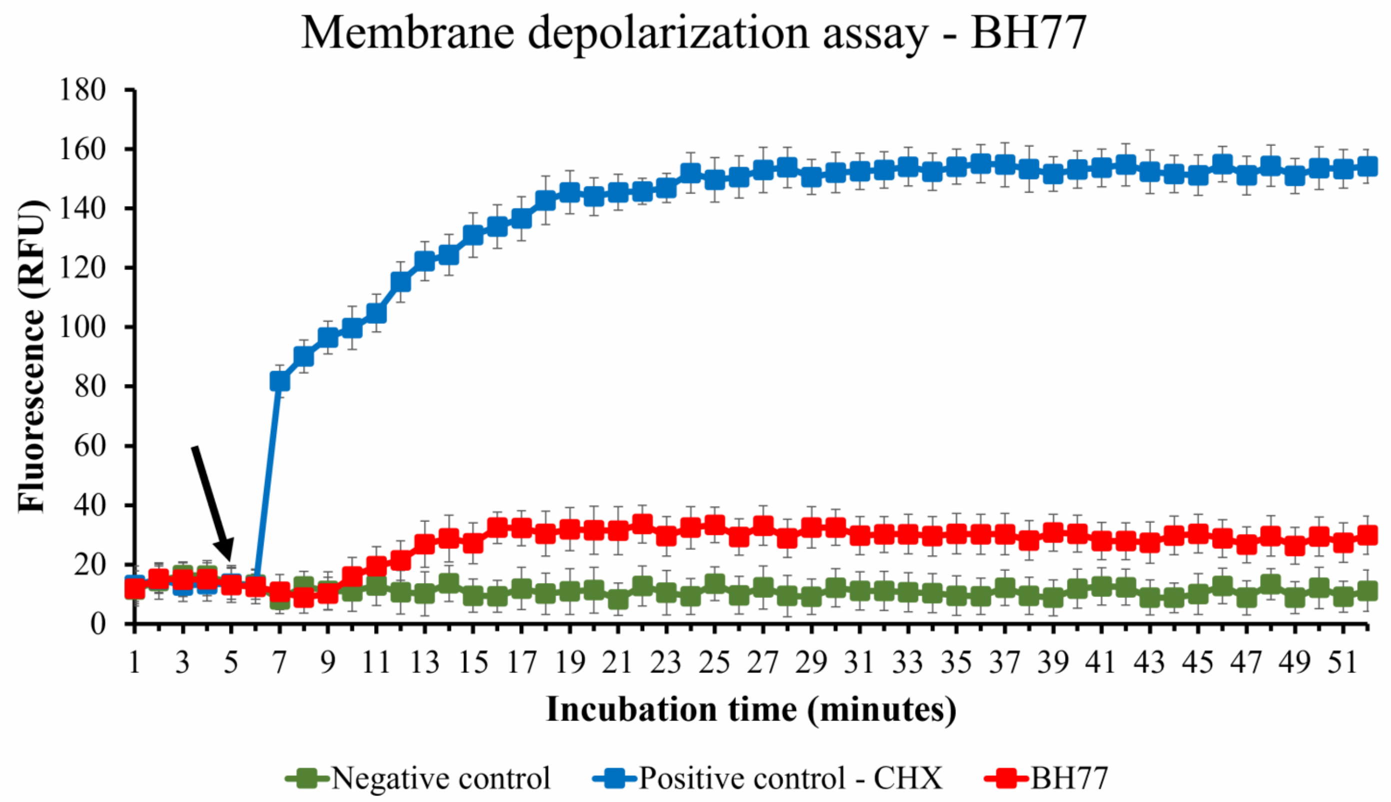

Fig. S3 $DiSC_3(5)$-based kinetic fluorescence measurement for detection of the impact of BH77 on membrane depolarization in *Staphylococcus aureus*. Methicillin-resistant *Staphylococcus aureus* (ATCC 43300) was stained with 0.5 μM $DiSC_3(5)$. After 15 min incubation period, the fluorescence signal (λEx = 620, λEm = 680) was continuously monitored. After 5 min of measurement (black arrow), BH77, and positive control represented by chlorhexidine (CHX), in final concentrations corresponding to minimum bactericidal concentrations, were added. Graph depicts the mean of eight replicates and standard deviation.

A. HepG2 cells

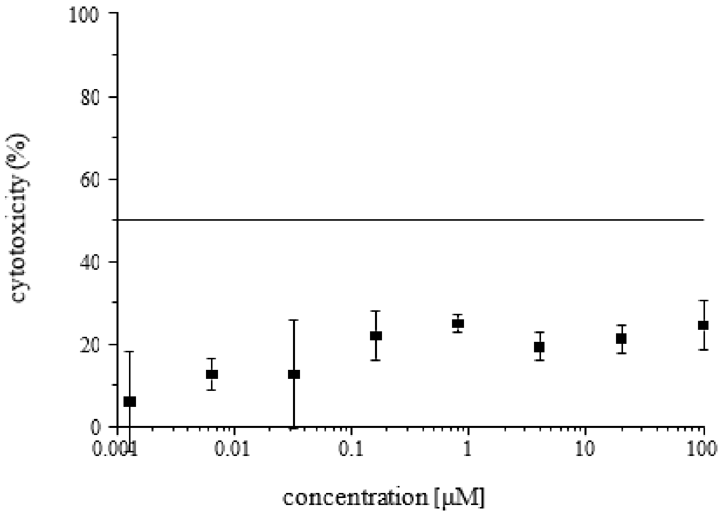

B. MonoMac6 cells

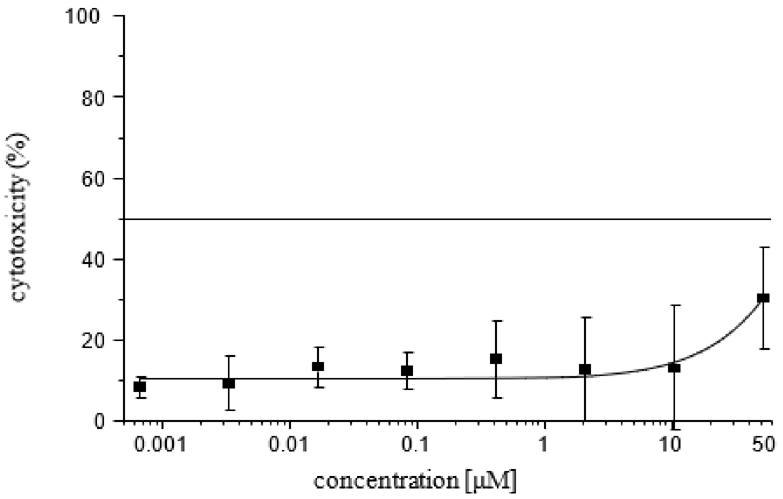

Fig. S4 *In vitro* cytotoxic effect of the tested compound, BH77. Two human cell lines were employed, namely HepG2 (A) and MonoMac6 (B). Different incubation concentrations (0.00128 – 100 µM for HepG2 (A) and 0.00064 – 50 for MonoMac6 (B)) were used in assays.

The curves were defined using MicrocalTM Origin 1 software (version 7.6; OriginLab, Northampton, MA, USA): cytotoxicity (%) was plotted as a function of concentration.

METHODS:

S1.1 Basic screening of antibacterial activity

The microdilution broth method was performed according to EUCAST (The European Committee on Antimicrobial Susceptibility Testing) instructions [S1], with slight modification. Eight tested bacterial strains were purchased from the Czech Collection of Microorganisms (CCM, Brno, Czech Republic) or the German Collection of Microorganisms and Cell Cultures (DSM, Braunschweig, Germany). Namely, four Gram-positive reference strains, *Staphylococcus aureus* subsp. *aureus* CCM 4223 (ATCC 29213), *Staphylococcus aureus* subsp. *aureus* methicillin-resistant (MRSA) CCM 4750 (ATCC 43300), *Staphylococcus epidermidis* CCM 4418 (ATCC 12228), *Enterococcus faecalis* CCM 4224 (ATCC 29212), and four Gram-negative reference strains, *Escherichia coli* CCM 3954 (ATCC 25922), *Klebsiella pneumoniae* CCM 4415 (ATCC 10031), *Acinetobacter baumannii* DSM 30007 (ATCC 19606), *Pseudomonas aeruginosa* CCM 3955 (ATCC 27853) were included in the basic antibacterial activity screening.

Briefly, the cultivation was done in Cation-adjusted Mueller-Hinton broth (CAMHB, M-H 2 Broth, Sigma-Aldrich, USA) at 35±2°C. Tested compound and selected antibiotics, used as internal quality controls were dissolved in DMSO (Sigma-Aldrich, USA) to produce stock solutions. The final concentration range of BH77 from 3.906 μM to 250 μM in CAMHB, with the final concentration of DMSO, corresponding to 1% DMSO (*v*/*v*), was prepared. Positive (microbe in CAMHB with final 1% DMSO, *v*/*v*), negative (CAMHB, 1% DMSO, *v*/*v*) controls, and internal quality standards (ciprofloxacin, gentamicin, and vancomycin; Sigma-Aldrich, USA) were involved in assays. Antibacterial activity, expressed as minimum inhibitory concentration (MIC, reported in μM), was evaluated after 24 and 48 h of static incubation in the dark and humidified atmosphere at 35±2°C. Visual inspection and

spectrophotometric measurement (530 nm, Synergy HTX Multi-Mode Microplate reader, BioTek, USA) were used for MIC endpoint evaluation.

S1.2 *In silico* prediction of physicochemical and structural parameters

Drug-likeness of the compound BH77 in preclinical development was assessed using Lipinski's rule of five [S2] based on molecular weight, lipophilicity (log*P*) and number of hydrogen bond donors and acceptors. In addition, prediction of gastrointestinal absorption and elimination of a possibility of having pan assay interference compound was also included. All these parameters and predictions were obtained using the freely available SwissADME predictor (http://www.swissadme.ch/index.php).

S1.3 Determination of bacteriostatic vs. bactericidal activity

To distinguish between the bactericidal or bacteriostatic activity of BH77, the strain MRSA ATCC 43300, microdilution method and subsequently spread plate technique for colony forming units (CFU) evaluation were employed. Solutions of BH77 in CAMHB with a final concentration range from 3.906 to 250 μM were prepared according to the procedure described above (section S1.1). After incubation for 24 hours, the MIC was evaluated and the representative aliquot from all wells where the growth inhibition was registered was taken, serially diluted, seeded, and subcultured on Mueller-Hinton agar for 24 hours in a humid atmosphere, at 35±2°C. Similarly, the initial bacterial inoculum was processed. After incubation, the number of CFU/ml was calculated, and MBC was evaluated. MBC is defined as the lowest concentration of antimicrobial agent that leads to the reduction of initial bacterial inoculum viability in ≥99.9%. The antibacterial agent is usually regarded as bactericidal if the MBC is not higher than four times the MIC.

S1.4 Evaluation of *in vitro* pharmacodynamic interaction

For the purpose of *in vitro* pharmacodynamic study of the interaction of BH77 with staphylococci, three selected bacterial strains, reference MRSA ATCC 43300, reference VRSA CCM 1767, and clinical isolate strain VRSA, ID No. 203/19, were chosen. The microdilution method mentioned above (section S1.1) was employed, and at the point of interest (every 30 minutes) during incubation for 20 hours at 36.8 °C, the optical density in the Bioscreen C instrument (540 nm, Oy Growth Curves, Finland) was measured.

S1.5 Determination of mechanism of action by macromolecular biosynthesis assay

Potential mechanism of action was investigated by method based on incorporation of [^{3}H] radioactively labelled biomolecules according to procedure described in literature [S3] with slight modifications. Strain used for screening was methicillin-resistant *Staphylococcus aureus* subsp. *aureus* CCM 4750, ATCC 43300. Four biosynthetic pathways were investigated using four macromolecules, namely *N*-acetylglucosamine for peptidoglycan synthesis, uridine for RNA synthesis, thymidine for DNA synthesis, and leucine for protein synthesis. Five standards with known mechanism of action were included (vancomycin – peptidoglycan; rifampicin – RNA; ciprofloxacin – DNA; chloramphenicol (CHL) – protein synthesis; chlorhexidine (CHX) – positive control). Results were read on the basis of radioactivity measured as counts per minute (cpm) with liquid scintillation counter TRI-CARB 2900TR (Perkin Elmer, USA) and compared to positive growth control (bacterial suspension without antibacterial compound).

Briefly, MRSA ATCC 43300 grown on Tryptic Soy agar (Sigma-Aldrich, USA) was transferred into Tryptic Soy Broth (TSB, Sigma-Aldrich, USA) and cultured overnight. Completely defined medium (CDM)/CDM-Leu (for determination of protein synthesis) prepared according to literature was used for preparation of log-phase culture of MRSA

(approx. 2×10^7 CFU). TSB culture was diluted with these media in ratio 1:100 and cultured at 37°C for another 5 hours. 0.9 ml of suspensions were transferred into pre-warmed glass tubes (14 tubes for 1 macromolecule). All antimicrobials (vancomycin – 8 mg/l, rifampicin – 0.064 mg/l, ciprofloxacin – 1 mg/l, chloramphenicol – 64 mg/l, chlorhexidine – 4 mg/l, BH-77 – 250 mg/l) were added at concentration equal to their 4×MIC and mixed thoroughly. All standards were purchased from Sigma-Aldrich (Sigma-Aldrich, USA). Untreated controls were incubated with adequate volume of DMSO (Sigma-Aldrich, USA). [^{3}H]-labelled precursors (*N*-acetylglucosamine – 0.1 μCi/ml, uridine – 1 μCi/ml, thymidine – 1 μCi/ml, leucine – 3 μCi/ml; Hartmann Analytic, Germany) were immediately added to corresponding tubes. Another incubation at 37°C for 2 hours followed. 0.5 ml aliquots were transferred into 2 ml ultracentrifuge tubes containing 1 ml of ice-cold 10% trichloroacetic acid (TCA, Sigma-Aldrich, USA), mixed thoroughly and placed on ice for at least 1.5 hours to facilitate the precipitation. The precipitates were then washed once with 0.5 ml of 5% TCA/1.5 M NaCl followed by one-time washing with 0.5 ml of 5% TCA to get rid of free precursors. After the second wash, samples were solubilised with 0.5 ml of 0.1% SDS/0.1 M NaOH by vortexing at room temperature. The solubilised precipitates were transferred into scintillation tubes and thoroughly mixed with 2 ml of scintillation cocktail (Sigma-Aldrich, USA). The incorporated radioactivity was measured in counts per minute using liquid scintillation analyser TRI-CARB 2900TR (Perkin Elmer, USA) and the results were expressed as percentage of untreated controls. Data were analysed using GraphPad Prism 8.0 software (GraphPad Software, USA). One-way analysis of variance (ANOVA) was used to determine the statistical significance (P) of differences in this *in vitro* assay.

S1.6 Determination of the impact of BH77 on staphylococcal cytoplasmic membranes

To detect the possible action of BH77 on the bacterial cytoplasmic membrane (membrane depolarisation), the fluorometric measurement of membrane potential using a voltage-sensitive dye, $DiSC_3(5)$, was employed. The assay was carried out using the reference strains, MRSA ATCC 43300, and chlorhexidine dihydrochloride (CHX) as the positive control.

Briefly, the MRSA strain was resuspended in CAMHB and cultivated to the exponential (mid-log) phase. The bacteria were compacted by centrifugation (10 000×g, for 10 min at 24°C), washed, resuspended in 5 mM HEPES, 5 mM glucose, pH 7.2, and finally diluted to optical density (O.D.) 0.5 McFarland units. The voltage-sensitive dye $DiSC_3(5)$ was then added to a final concentration of 0.5 μM (1% DMSO, *v*/*v*). After a short incubation period (15 min), the bacterial suspension was transferred to a white polystyrene 96-well plate (200 μl/per well), and fluorescence quenching (λEx = 620, λEm = 680, Synergy HTX Multi-Mode Microplate reader, BioTek, USA) was monitored for 5 minutes (until a stable baseline was obtained). Depolarising and the tested compounds were added to the wells in hexaplicates (with a final concentration of 1% DMSO, *v*/*v*). CHX at a final concentration of MBC (4 mg/l, 1% DMSO, *v*/*v*) was added to the wells serving as the positive control. The negative control was represented by untreated, stained bacterial cells in a HEPES buffer (1% DMSO, *v*/*v*). The impact of BH77 on the cytoplasmic membrane was revealed by the addition of BH77 at a final concentration of MBC (62.5 μM). The plate was quickly replaced back into the reader to continuously monitor fluorescence every 1 min for the next 45 min.

S1.7 Evaluation of BH77 *in vitro* cytotoxicity

Human hepatocellular carcinoma cells HepG2 (ATCC HB-8065) were cultured in Dulbecco´s Modified Eagles Medium High Glucose (denoted DMEM High Glucose; Merck, USA) supplemented with fetal bovine serum (10 %), Non-Essential Amino Acids (10 %) and penicillin/streptomycin (10 %). Human Leukemia MonoMac6 cells (ACC124; Deutsche

Sammlung von Mikroorganismen and Zellkulturen GmbH, Braunschweig, Germany) were cultured in RPMI-1640 medium (Lonza, Basel, Switzerland) containing 10% fetal bovine serum, 2 mM L-glutamine, and 1% streptomycin-penicillin (from 10,000 units of benzylpenicillin and 10 mg of streptomycin/ml; Gibco, Thermo Fisher Scientific, Waltham, MA, USA). Both cell lines were cultured at 37 °C, 5% CO_2 in humidified atmosphere and grown to confluence and were plated into 96-well plate with initial cell number of $1.0x10^4$ (HepG2) or 5.0×10^3 (MonoMac6) in 100 µl per well. Cells were seeded during the exponential growth phase. After 24 h incubation at 37 °C, cells were treated with the tested compounds in 100 µL (HepG2) or 200 µL (MonoMac6) final volume containing 1% DMSO (*v*/*v*). Cells were incubated with the compounds at 0.00128 – 100 or 0.00064 – 50 µM concentration range (for HepG2 and MonoMac6 , respectively) of the title compound for 24 h. Controls were treated with serum free medium (DMEM High Glucose/RPMI-1640) only or with DMSO (1% *v*/*v*) at 37 °C for 24 h.

After incubation, the MonoMac6 cells were washed twice with serum free RPMI-1640 (centrifugation: 1000 rpm, 5 min). Then, 3-(4,5-dimethylthiazol-2-yl)-2,5-diphenyltetrazolium bromide (MTT)-assay was carried out immediately after the washing step following the overnight treatment. MTT (45 µl, 2 mg/ml) was added to each well, which was reduced to insoluble purple formazan crystals within the living cells. After 3.5 hours of incubation at 37 °C the cells were centrifuged (200 rpm, 5 min) and supernatant was removed. The formazan crystals were dissolved in 100 µl of DMSO and the optical density of the samples was measured at 540 and 620 nm, employing an ELISA Reader instrument (iEMS Reader, Labsystems, Helsinki, Finland).

For HepG2 cells, when 24h incubation was over, the reagent from the kit CellTiter 96 AQueous One Solution Cell Proliferation Assay (CellTiter 96; PROMEGA, Fitchburg, USA)

was added. After 2h incubation at 37 °C, absorbance in each sample well was recorded at 490 nm (TECAN, Infinita M200, Austria).

OD_{620} values were subtracted from OD_{540} values and the percent of cytotoxicity was calculated using equation: "cytotoxic effect (%) = $[1 - (OD_{treated}/OD_{control})] \times 100$" (where $OD_{treated}$ and $OD_{control}$ correspond to the optical densities of the treated and the control cells, respectively). For each compound, at least two independent experiments were carried out with four parallels. The 50% inhibitory concentration (IC_{50}) values were determined from the dose-response curves. The curves were defined using MicrocalTM Origin 1 software (version 7.6; OriginLab, Northampton, MA, USA): cytotoxicity (%) was plotted as a function of concentration, fitted to a sigmoidal curve and, based on this curve, the half maximal inhibitory concentration value was determined representing the concentration of a compound required for 50% inhibition.

REFERENCES

S1. European Committee for Antimicrobial Susceptibility Testing (EUCAST) of the European Society for Clinical Microbiology and Infectious Diseases (ESCMID). 2003. EUCAST Discussion Document E. Dis 5.1: Determination of Minimum Inhibitory Concentrations (Mics) of Antibacterial Agents by Broth Dilution. Clin Microbiol Infect 9:1-7.

S2. Lipinski CA. 2004. Lead- and drug-like compounds: the rule-of-five revolution. Drug Discov. Today Technol 1:337–341.

S3. Nowakowska J, Griesser HJ, Textor M, Landmann R, Khanna N. 2013. Antimicrobial properties of 8-hydroxyserrulat-14-en-19-oic acid for treatment of implant-associated infections. Antimicrob Agents Chemother 57(1):333-342.
